# Splicing Analysis of Exonic TSC1 and TSC2 Gene Variants Causing Tuberous Sclerosis Complex

**DOI:** 10.1155/humu/1497712

**Published:** 2025-04-01

**Authors:** Qingqing You, Jingwei Liu, Ran Zhang, Zhi Wang, Bingying Zhang, Wencong Guo, Ning Xu, Irene Bottillo, Leping Shao

**Affiliations:** ^1^Department of Nephrology, Qingdao Municipal Hospital (Group), Qingdao Hospital of University of Health and Rehabilitation Sciences, Qingdao, China; ^2^Department of Cardiac Surgery, Qingdao Municipal Hospital (Group), Qingdao Hospital of University of Health and Rehabilitation Sciences, Qingdao, China; ^3^School of Clinical Medicine, Shandong Second Medical University, Weifang, China; ^4^Institute of Nephrology, Zhongda Hospital, Southeast University School of Medicine, Nanjing, China; ^5^Division of Medical Genetics, Department of Experimental Medicine, San Camillo-Forlanini Hospital, Sapienza University, Rome, Italy; ^6^Department of Nephrology, (Fujian Provincial Clinical Research Center for Glomerular Nephritis), The First Affiliated Hospital of Xiamen University, School of Medicine, Xiamen University, Xiamen, China

**Keywords:** exon variants, minigene assay, splicing, TSC

## Abstract

Tuberous sclerosis complex (TSC) is characterized by abnormalities in cell proliferation and migration, leading to the development of hamartomas, benign tumors, or malignant cancers, affecting both the skin and brain, as well as potentially impacting the heart, kidneys, lungs, and eyes, with varying patterns of involvement over a lifetime. It is primarily caused by mutations in the TSC1 and TSC2 genes. Aberrant splicing is a crucial factor in hereditary diseases. Alternative splicing is a key mechanism for expanding the diversity of the human proteome. Mutations disrupting canonical splice sites or splicing regulatory elements impede the utilization of splice sites, leading to exon skipping and intron retention. We comprehensively analyzed missense and nonsense mutations of TSC1 and TSC2 genes using bioinformatics tools and identified 10 candidate mutations affecting pre-mRNA splicing through minigene analysis. Mutations in TSC genes can lead to partial or complete exon skipping and/or intron retention through complex mechanisms. This study emphasizes the importance of evaluating their roles in the splicing of suspected pathogenic variants in TSC.

## 1. Introduction

Tuberous sclerosis complex (TSC) is an autosomal dominant genetic disease caused by loss-of-function mutations in the TSC1 or TSC2 genes, which can affect multiple organ systems such as the brain, kidney, and skin. Generally speaking, individuals with TSC2 gene mutations tend to have a more severe phenotype compared to those with TSC1 gene mutations [1, 2].

An increasing number of studies show that splicing mutation plays an important role in human genetic diseases [3]. It is well known that the removal of introns from pre-mRNA and the joining of exons is a critical aspect of gene expression [4]. This process is finely controlled by a broad array of *cis*-acting elements that are responsible for splicing efficiency [5]. And these elements are recognized by specific splicing repressors and activators that help to properly carry out the splicing process [6]. Sequence variations occurring in exons or introns may affect the correct processivity of the mRNA by disrupting the splice site, exonic splicing enhancers/exonic splicing silencers (ESEs/ESSs), and intronic splicing enhancers/intronic splicing silencers (ISEs/ISSs) [7].

Mutations in the TSC1 and TSC2 genes encompass a range of types, including missense/nonsense variants, splice site mutations, and small deletions/insertions; approximately 10.67%~12.41% are splicing variants (Human Gene Mutation Database (HGMD) Professional 2023.4) among them. In recent years, with advances in next-generation sequencing and whole-gene sequencing, an increasing number of TSC-gene mutations have been identified; nevertheless, the impact of these mutations on the pre-mRNA splicing process has been widely disregarded [8]. The purpose of the present study was to evaluate the effects of a set of exon mutations in the TSC1 and TSC2 genes, which cause TSC, on pre-mRNA splicing using a minigene-based approach.

## 2. Materials and Methods

### 2.1. Bioinformatic Prediction and Selection of Variants

All TSC1 and TSC2 variants were selected from the HGMD (Professional 2023.4). In order to select mutations with potential effects on pre-mRNA processing, all variants were predicted by bioinformatics analysis software. We used online software SpliceAI (https://spliceailookup.broadinstitute.org/) and BDGP (https://www.fruitfly.org/) to evaluate the impact of variants on consensus 5⁣′ donor site (DS) or 3⁣′ acceptor site (AS). The HExoSplice (http://bioinfo.univ-rouen.fr/HExoSplice/) was designed to predict the effect of mutation on potential splicing regulatory elements (SREs) such as ESEs and ESSs. Besides, we used VulExMap (https://vulexmap.compbio.sdu.dk/) to predict whether an exon is vulnerable to aberrant splicing [9], and DeepCLIP (https://deepclip-web.compbio.sdu.dk/) was used to analyze RNA-binding proteins involved in exon regulation [10].

Variants that met the following criteria were selected for further minigene splicing assays: close to the 5⁣′ or 3⁣′ end of the exon and elimination of ESEs or creation of ESSs. In addition, for SpliceAI, three different cutoffs were proposed: 0.2 (high sensitivity cut-off), 0.5 (usually recommended cutoff), and 0.8 (high specificity cutoff). Therefore, variants with scores greater than the usually recommended cutoff (0.5) were also included in this study.

### 2.2. Minigene Constructions and Mutagenesis

For each variant identified, a minigene assay was performed as previously described [11]. Genomic DNA was extracted from healthy human blood using a DNA purification kit (Promega Corporation). In the in vitro splicing experiment, the fragment consisting of the target exon was amplified by specific primers (Table S1). The fragment with the wild-type (WT) target gene was cloned into the splicing vector pSPL3 to form the WT plasmid (Figure S1). Variants were introduced into the WT plasmid using the QuikChange II Site-Directed Mutagenesis Kit (Stratagene, La Jolla, CA). Mutagenic primers were designed using the PP5 software, as detailed in Table S2. All constructs were confirmed by sequencing (Figure S2).

### 2.3. Splicing Functional Assays

HEK 293T and Hela cells were transiently transfected with either WT or mutated plasmid using Lipofectamine 2000 (Invitrogen, Carlsbad, CA, United States). In order to prevent nonsense-mediated RNA degradation (NMD), the nonsense variants were cocultured with puromycin for 6 h before RNA extraction [4]. After 48 h, an RNeasy isolation reagent (Vazyme) was used to extract total RNA, which was converted into cDNA by RT-PCR using the reverse transcription kit (Evo M-MLV Plus cDNA Synthesis kit). The resulting cDNA was amplified by PCR; then, PCR products were identified by 1.5% agarose gel, and each band was quantified by ImageJ software. The percentage of abnormal transcripts (%) = (aberrant band/all band) × 100. Statistical analysis was performed using GraphPad Prism (Version 6.02, United States); ⁣^∗^*p* < 0.05 was considered statistically significant. All transcripts were analyzed by DNA Sanger sequencing (Figures S3–S9).

## 3. Results

**Figure 1:**
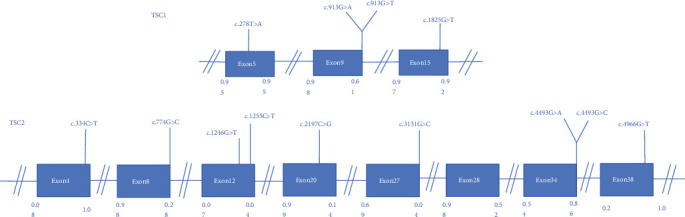
Position of candidate variants in TSC1 and TSC2 gene. Blue boxes and black lines between them represent the coding exons and intron sequences, respectively. Their sizes are not proportional. The BDGP scores of DS and AS are shown at the bottom.

According to the screening criteria in the materials and methods, we finally selected the 15 candidate variants from TSC1 and TSC2 genes, and minigenes were generated by site-directed mutagenesis based on the corresponding pSPL3-WT minigene. Variants c.4662G>T and c.4846C>T were not verifiable due to technical reasons. The characteristics of TSC1 and TSC2 gene variants chosen for this study, along with the results of bioinformatics analysis, are presented in Figure 1 and Table 1.

### 3.1. In Silico Analysis of Variations

In our study, bioinformatic predictions indicate that the mechanisms by which these 13 variants affect splicing are not always singular. Variants c.913G>A and c.913G>T of the TSC1 gene and variants c.774G>C, c.1246G>T, c.4493G>A, c.4493G>C, and c.4966G>T of the TSC2 gene are predicted to activate cryptic splice sites. Variants c.278T>A of the TSC1 gene and c.334C>T and c.1255C>T of the TSC2 gene are predicted to create new splice sites. Variants c.1825G>T of the TSC1 gene and c.2197C>G and c.3131G>C of the TSC2 gene are predicted to primarily affect the proportion of ESEs/ESSs, while variants c.334C>T, c.1246G>T, and c.4966G>T of the TSC2 gene are predicted to not only affect splice sites but also alter the ratio of regulatory elements (Table 1). VulExMap predicts that the TSC1 gene has eight vulnerable exons (8/23), and the TSC2 gene has three vulnerable exons (3/42). In this study, only two mutations arise from vulnerable exons: c.278T>A, found in Exon 5 of the TSC1 gene, and c.334C>T, located in the last 3 bp of Exon 4 in the TSC2 gene. This low ratio may be related to the fact that most of the mutations selected in our experiment are located within the first or last 3 bp of the exons (Figure S10). DeepCLIP predicts that the variants c.1246G>T and c.4966G>T in the TSC2 gene significantly reduce the ability to bind SR proteins such as SRSF1 and SRSF9. Variants c.278T>A and c.2197C>G increase the binding score for the theoretical repressor proteins HNRNPA1 and HNRNPL, which could explain their higher splicing impact (Table S3). In addition, protein-level predictive analysis was performed of 11 missense variants among these alterations (Table S4).

### 3.2. Minigene Analysis

**Figure 2:**
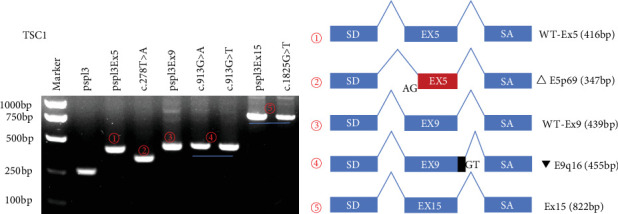
Agarose gel electrophoresis of the TSC1 gene and schematic representation of splicing events and transcripts generated from the wild type and mutant. The electrophoresis results are presented on (a), while splicing events and transcripts are on (b). Lane 1: marker; Lane 2: pSPL3 (263 bp); Lane 3: pSPL3-Ex5 (416 bp); Lane 4: c.278T>A (347 bp); Lane 5: pSPL3-Ex9 (439 bp); Lane 6: c.913G>A (455 bp); Lane 7: c.913G>T (455 bp); Lane 8: pSPL3-Ex15 (822 bp); Lane 9: c.1825G>T (822 bp). Exons and splicing reaction exons are represented by blue boxes and lines; skipped exons are shown in red and intron in black.

**Figure 3:**
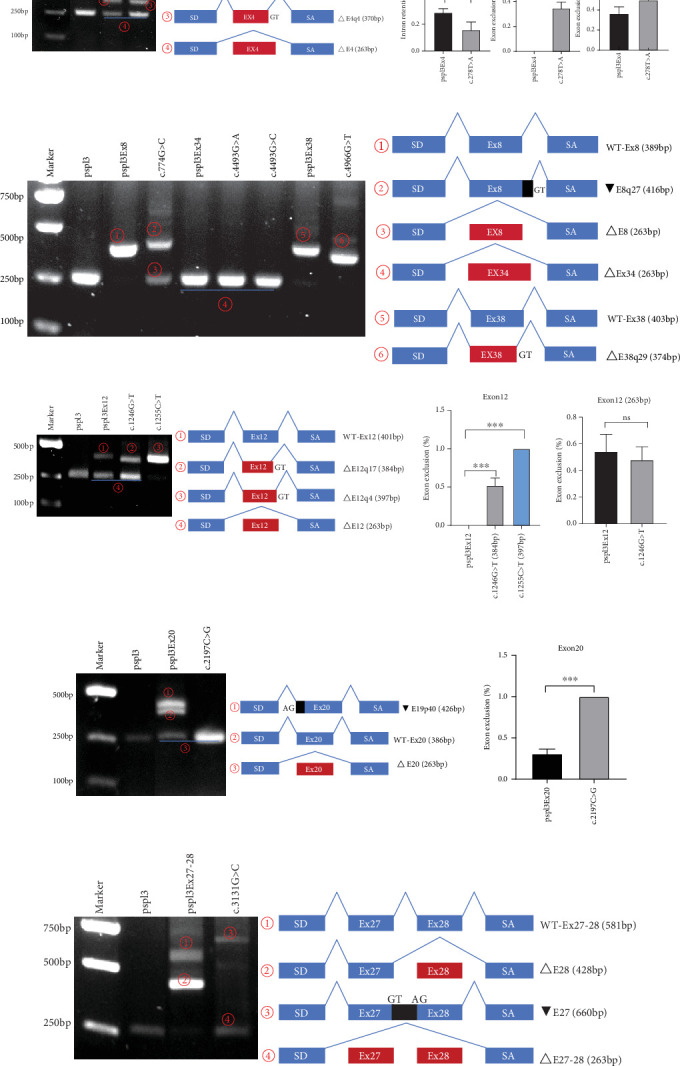
Electrophoresis results and transcription analysis of wild-type and mutant minigenes of the TSC2 gene. (a) Agarose gel electrophoresis, splicing events, and statistical analysis of RT-PCR products of Exon 4. Lane 1: marker; Lane 2: pSPL3; Lane 3: pSPL3-Ex4; Lane 4: c.334C>T. (b) Agarose gel electrophoresis and splicing events of RT-PCR products of Exon 8, Exon 34, and Exon 38. Lane 1: marker; Lane 2: pSPL3; Lane 3: pSPL3-Ex8 (389 bp); Lane 4: c.774G>C; Lane 5: pSPL3-Ex34 (263 bp); Lane 6: c.4493G>A (263 bp); Lane 7: c.4493G>C (263 bp); Lane 8: pSPL3-Ex38 (403 bp); Lane 9: c.4966G>T (374 bp). (c) Agarose gel electrophoresis, splicing events, and statistical analysis of RT-PCR products of Exon 12. Lane 1: marker; Lane 2: pSPL3; Lane 3: pSPL3-Ex12; Lane 4: c.1246G>T; Lane 5: c.1255C>T (397 bp). (d) Agarose gel electrophoresis, splicing events, and statistical analysis of RT-PCR products of Exon 20. Lane 1: marker; Lane 2: pSPL3; Lane 3: pSPL3-Ex20; Lane 4: c.2197C>G (263 bp). (e) Agarose gel electrophoresis, splicing events, and statistical analysis of RT-PCR products of Exons 27–28. Lane 1: marker; Lane 2: pSPL3; Lane 3: pSPL3-Ex27-28; Lane 4: c.3131G>C. In (a), (c), and (d), the splicing events are shown in the middle; the rightmost is the statistical analysis of RT-PCR products. Abnormal transcripts (%) = (abnormal band/all band) × 100. Error bars represent SEM (*n* = 3). ⁣^∗^*p* < 0.05, ⁣^∗∗^*p* < 0.01, and ⁣^∗∗∗^*p* < 0.001. Exons and splicing reaction exons are represented by blue boxes and lines; skipped exons are shown in red and intron in black. Full gel photographs for minigene assays are shown in Figures S12 and S13.

**Figure 4:**
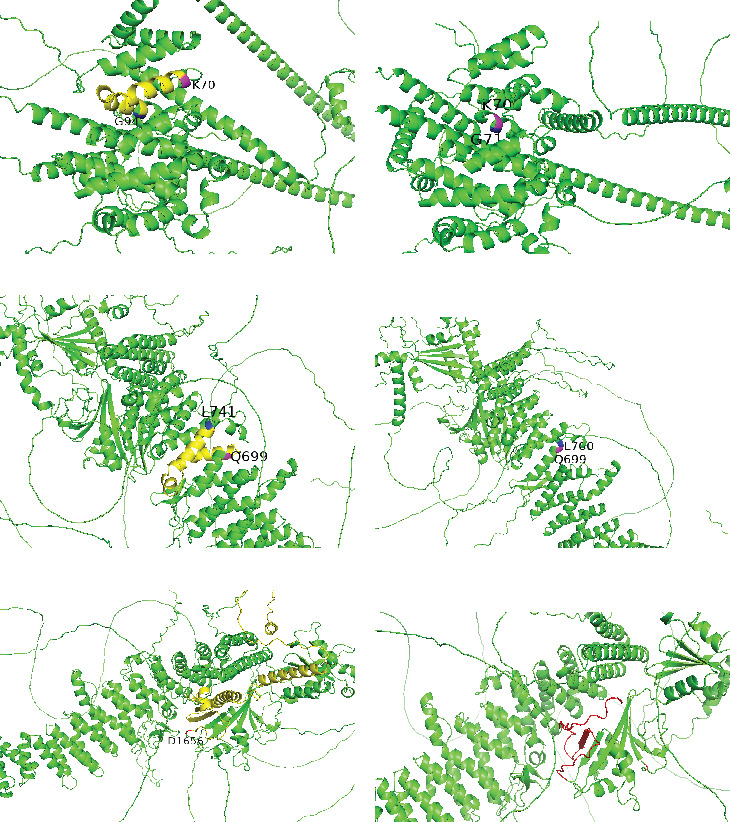
Structural predictions for TSC variants. Cartoon representation of wild-type TSC in (a), (c), and (e) and mutants in (b), (d), and (f). (a) WT TSC1, K70 is shown in magenta, G94 is shown in blue, and the amino acids between K70 and G94 are shown in yellow. (b) The variant c.278T>A results in a loss of 23 amino acids from K70 to G94 in TSC1-NTD. (c) WT TSC2, Q699 is shown in magenta, L741 is shown in blue, and the amino acids between Q699 and L741 are shown in yellow. (d) The variant c.2197C>G results in the deletion of 41 amino acids from Q699 to L741 in the tuberin-type domain. (e) WT TSC2, D1656 is shown in red, and the subsequent amino acids are shown in yellow. (f) The variant c.4966G>T results in the formation of a truncated protein in the GAP domain (the truncated protein is shown in red).

The minigene assays showed a significant splicing impact for 10/13 candidate variants, including exon skipping (5/10), intronic retention (5/10), and exon deletion (3/10). Simultaneously, the variants may generate multiple forms of abnormal transcripts (Figures 2 and 3). Seven transcripts were predicted to introduce premature termination codons (PTCs) due to frameshift, while eight transcripts still kept the reading frame. The consequences of aberrant splicing caused by the mutations and the impact on the protein level are shown in Table 2. We also utilized AlphaFold3 (https://alphafoldserver.com/) to model the effect of the variants on protein structure and visualized the results using PyMOL (Version 3.1) (Figure 4). All variants reported in this study were reclassified according to the principles of standards and guidelines recommended by the American College of Medical Genetics and Genomics (ACMG) [12] (Table 2).

### 3.3. Splicing Outcome of Variations of TSC1 Gene

Except for variant c.1825G>T, three of the variants in the TSC1 gene affect splicing. RT-PCR results revealed a band of 416 bp in size for the WT (pSPL3 Ex5) minigene and a band of 347 bp for the variant c.278T>A (Figure 2). Subsequent sequencing confirmed that the 416 bp band contained Exon 5, while the variant generated a novel splice AS, resulting in a 69 bp deletion at the 5⁣′ end of Exon 5 (Figure S3A).

Both the WT (pSPL3-Ex9) minigene and variants c.913G>A and c.913G>T yielded a single electrophoretic band. The transcript produced by WT was a 439 bp transcript containing Exon 9, while the fragments produced by c.913G>A and c.913G>T were both 455 bp in size. Sequencing confirmed the partial retention of Intron 9 (16 bp) in addition to Exon 9 (Figure 2 and Figure S3B).

### 3.4. Splicing Outcome of Variations of TSC2 Gene

Apart from c.4493G>A and c.4493G>C, seven variants in TSC2 affect splicing. The minigene assays showed the WT (pSPL3-Ex 4) generated three transcripts: the largest transcript is 464 bp in size, which includes Exon 4 and 90 bp upstream Intron 4; the middle transcript is the normal spliced transcript, 374 bp in size; and the last one was skipping Exon 4 (Figure 3a). The variant c.334C>T also yields three products, yet it lacks normally spliced ones. Besides the same two transcripts involving partial Intron 4 and Exon 4 skipping, the variant also generates a new DS, resulting in the deletion of the last four bases at the 3⁣′ end of Exon 4 (Figure 3a and Figure S4).

The lane of WT (pSPL3-Ex8) contained only a 389 bp fragment of Exon 8, whereas c.774G>C generated two different fragments of 416 and 263 bp, respectively. The fragments of 416 bp size included an upstream 27 bp fragment of Intron 8, while the other fragment involved skipping of Exon 8 (Figure 3b and Figure S5).

The minigene assay results identified that cDNA products generated by the mutant and WT (pSPL3-Ex 38) minigenes were different. The band of WT carried one fragment of 403 bp that contained Exon 38, whereas sequencing results indicated that variant c.4966G>T activated a cryptic DS, resulting in the deletion of 29 bp of the 3⁣′ end of Exon 38 (Figure 3 and Figure S6).

The minigene experiment validated that two bands of WT (pSPL3-Ex12) were 401 and 263 bp, respectively, representing normal splicing and Exon 12 skipping. Variant c.1246G>T also generated two different transcripts, including a larger (384 bp) and a smaller (263 bp) one (Figure 3c). Sequencing results revealed that the larger one contained a partial Exon 12 lacking 17 bp at the 3⁣′ end, while the smaller one exhibited Exon 12 skipping. Additionally, variant c.1255C>T only generated one aberrant band of 397 bp, with sequencing confirming the deletion of the last four bases of Exon 12 (Figure S7).

The WT (pSPL3-Ex20) minigene generated three different fragments of sizes 426, 386, and 263 bp, corresponding to the transcripts containing a 40 bp downstream of Intron 19, normal splicing transcript, and the transcript with Exon 20 skipping. In contrast, variant c.2197C>G just showed one fragment lacking Exon 20 (Figure 3d and Figure S8).

The WT minigene composed of Exon 27 and Exon 28 could generate two transcripts. Direct sequencing revealed that the 581 bp transcript contained both Exon 27 and Exon 28, while the smaller one (428 bp) only included Exon 27. The two products generated by variant c.3131G>C were completely different from the WT. The 660 bp band not only contained Exon 27 and Exon 28 but also caused the retention of Intron 27. The smaller transcript simultaneously skipped Exon 27 and Exon 28 (Figure 3e and Figure S9).

### 3.5. Classification of DNA Variants

It is well known that the mechanism of TSC disease is loss of function, so evidence of PVS1 is applicable to confirm variants generating abnormal transcripts in the minigene experiments, including frameshift mutations and single or multiexon deletions, while the weight of PVS1 is downgraded based on the structural features of critical regions and the size/position of in-frame mutations. All reported substitutions fulfilled the criterion PM2 because no allele frequency was reported in gnomAD. All variants are applicable to the PP4 criterion because the patient's phenotype or family history is highly specific for TSC with a single genetic etiology. We evaluate the pathogenicity of different transcription products from variants separately. The variant c.334C>T of the TSC2 gene produces three different transcripts; however, alternative splicing products exist among them. Hence, we assess only the abnormal transcripts (△E4q4) differing from the WT. The variant c.3131G>C of the TSC2 gene produces a transcript (△E27-28) that may result in partial loss of domains with unclear functionality, thus necessitating degradation to PVS1_m. We currently lack clarity on the influence of the ▼E8q27 transcript on protein outcomes generated by variant c.774G>C of the TSC2 gene. In summary, after validation through minigene experiments, nine variants previously classified as variants of uncertain significance (VUS) have been reclassified as pathogenic (Table 2).

## 4. Discussion

Alternative splicing is a crucial mechanism for gene regulation and for generating proteomic diversity [13]. Alterations in splicing may be one of the major mechanisms by which exon mutations cause phenotypic variation and human disease [14]. Many sequence variations affect disease risk; actually, they may have unexpected deleterious effects on the splicing and protein translation mechanisms [15]. The aberrant splicing of pre-mRNA due to the presence of point mutations, for example, nucleotide substitutions, that alter the consensus splicing regulatory sequences in a specific gene may lead to specific hereditary monogenic disorders. We conducted a comprehensive analysis of the effects of TSC-gene variants on pre-mRNA splicing using bioinformatics tools and minigene analysis.

### 4.1. Impact of Mutations on Splicing

In general, mutations in the canonical AS and DSs affect strongly conserved sequences that define exon–intron boundaries [6]. The research on splicing mutations indicated that the DS mutations were more prevalent than the AS variants (ratio 1.5:1) [16]. In the present study, among the 10 variants affecting splicing, eight variants were found to impact the DS.

Variants c.913G>A and c.913G>T of the TSC1 gene activate a cryptic DS in Intron 9, which is located at 16 bp downstream of the 3⁣′ end of Exon 9, resulting in the insertion of 16 bp of Intron 9. Au et al. [17] identified two missense variants for the same codon, G305 (p.G305R and p.G305W), in Exon 9 in two independent families with TSC, whereas the functional significance of these changes is not known. Two previous function-expressing studies verified that both variants of c.913G>A and c.913G>T were neutral and predicted them as more likely pathogenic splicing mutations, not missense mutations [18, 19]. Our minigene results confirmed their hypothesis.

Exon 8 of the TSC2 gene exhibits a notably weak DS score (BDGP score, 0.28), suggesting a high propensity for abnormal splicing. Variant c.774G>C, located at the last nucleotide of Exon 8, causes the weakening of this splice site (BDGP score, 0.28→NA) and may activate potential cryptic splice sites. It is worth noting that the score of the potential splicing site (27 nt upstream of Intron 8) is also not high (BDGP score, 0.15). The minigene experiment exhibits one product in higher abundance (▼E8q27 72.54%) and another in lower abundance (△E8 27.46%), indicating that in the mutated context, the cryptic splice site is used preferentially despite its lower raw score. The mutation was categorized as a missense variant in the literature [20], whereas our minigene experiment demonstrated it is a splicing variant.

Regarding Exon 4 in the TSC2 gene, three transcripts were observed in the WT minigene, demonstrating the complex alternative splicing of TSC2. Interestingly, the ratios of the three transcripts of the WT pspl3-Ex4 are consistent. This may be related to Exon 4 being a vulnerable exon (Figure S10), which is particularly susceptible to splicing mutations. Furthermore, BDGP predicts that the 5⁣′ DS score of Exon 4 (1.0) and the cryptic donor score 90 bp downstream of Exon 4 (0.98) are nearly identical, and the similar donor recognition capabilities could lead to a comparable ratio of products. The MT also exhibits complicated alternative splicing, with the main difference being the absence of the normal product, resulting in a new splicing product (△E4q4), primarily due to the emergence of a new DS. On the other hand, as López-Bigas et al. mentioned in their study, genes that have several alternative splicing forms are expected to have more regulatory sequences of the splicing process than genes not alternatively spliced, and these sequences could be a target for mutations [3]. Therefore, considering the predictions from HExoSplice and DeepCLIP, splicing regulatory sequences may also be involved in the mechanism by which variant c.334C>T causes complex splicing.

Likely, the same alternative splicing events also occur in Exon 12, Exon 20, and Exon 27. Variants c.1246G>T and c.1255C>T activate a cryptic splice site and generate a new splice site, respectively, resulting in transcripts different from the WT. Additionally, DeepCLIP predicts that variant c.1246G>T disrupts binding with SRSF1/SRSF7/SRSF9 and enhances binding with HNRNPL, which could explain its impact on alternative splicing. Similarly, WT-Ex20 produces three different transcripts, which are not only attributed to various SREs associated with alternative splicing but also to a strong cryptic splice AS located 40 nt upstream of the 5⁣′ end of Exon 20. Contrary to the generation of a new splice DS predicted by SpliceAI at the 24 nt downstream of Exon 20, the minigene demonstrates that c.2197C>G only produces a transcript with Exon 20 skipping. This may be linked to the formation of ESSs (CAGGTG/AGGTGT/GGTGTG), which increases the probability of the variant binding with the inhibitory proteins HNRNPA1 and HNRNPL.

Most interesting is variant c.3131G>C at the last position of Exon 27, which has a weak DS score (BDGP, 0.04→0.00). The MT minigene generated two completely distinct transcripts, one comprised of Exon 27–Intron 27–Exon 28, the other skipping Exon 27 and Exon 28. There are several explanations for the mechanism of the two-exon skipping in the previous literature [6, 21–23]. The variant c.3131G>C might lead to “splicing paralysis,” with slower removal of Intron 27 or fusion of Exon 27–Intron 27–Exon 28 into a single exon, resulting in both exons skipping and/or retention of Intron 27. Moreover, the DS score of Exon 27 is very low (BDGP, 0.04), and the spliceosome fails to recognize the 5⁣′ss of Exon 27 and the 3⁣′ss of Exon 28, causing skipping of Exons 27 and 28.

Dufner et al. [24] have previously demonstrated the impact of the variant c.4966G>T on splicing, where the variant activates an upstream cryptic 5⁣′ss, causing a frameshift and resulting in premature termination of the TSC2 open reading frame (ORF). Our experimental findings align with these results.

Finally, variants c.1825G>T, c.4493G>A, and c.4493G>C have a high biological score that can lead to aberrant splicing, but they were not significantly different from the WT in our minigene experiments. Although bioinformatics tools such as SpliceAI, BDGP, and HExoSplice provide valuable predictions, discrepancies between computational predictions and experimental outcomes can occur. The minigene assay result may be different from the in vivo splicing result and probably cannot completely reveal splicing change in carriers with the same splicing variant [8]. And the secondary structure of pre-mRNA molecules can have an enhancing or inhibitory effect on pre-mRNA splicing [25]. Moreover, the development of CRISPR/Cas9 gene editing and RNA-seq technologies [26–28] allows for a more comprehensive exploration of the splicing outcomes of gene mutations through the integration of minigene assays with these tools. These integrated approaches not only enhance our understanding of how mutations affect splicing but also provide more accurate predictions of their potential pathogenicity in vivo.

### 4.2. Consequence of Splicing and Influence on Protein Structure

**Figure 5:**
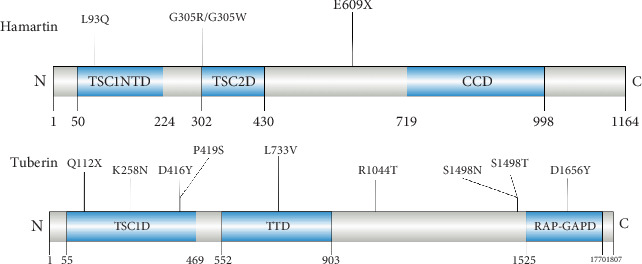
Biochemical structures of hamartin and tuberin and the positions of amino acid substitutions. TSC1NTD, TSC1N terminal domain; TSC2D, TSC2 binding domain; CCD, coiled-coil domain; TSC1D, TSC1 binding domain (hamartin binding domain); TTD, tuberin-type domain; RAP-GAPD, RAP-GAP domain.

TSC1 and TSC2 genes encode hamartin and tuberin, respectively. Hamartin and tuberin form a functional heterodimer that constitutively inhibits the mammalian target of rapamycin (mTOR) signaling pathway, helping regulate cellular proliferation [29]. TSC1 and TSC2 are both required for full TSC1-TSC2 activity. TSC1 and TSC2 form a stable complex through interactions between the N-terminal domain of TSC2 (aa. 1–900) and multiple regions of TSC1, which include the tuberin binding domain (TSC2D; aa. 302–420) and the coiled-coil domain close to the C-terminus of TSC1 (aa. 719–988) [30] (Figure 5).

Variants in critical domains of the TSC complex correlate with increased disease severity. In our study, two missense variants (c.913G>A and c.913G>T) in the TSC1 gene, three missense variants (c.774G>C, c.1246G>T, and c.1255C>T), and one nonsense variant (c.334C>T) in the TSC2 gene were found in the binding region of TSC1 and TSC2 (Figure 5). These variants affected the splicing of the TSC1 and TSC2 genes, resulting in changes to protein length or the formation of truncated proteins, which impacted the stability of the complex (Figure 4 and Figure S11). The most conserved region among the TSC1 protein is the N-terminal domain (TSC1-NTD), especially the *α*-helical “core” domain between amino acids 50 and 224, which is critical for the function and stability of TSC1. This region also anchors most of the disease-associated pathogenic TSC1 mutations identified [31, 32]. The variant c.278T>A is located inside the hydrophobic cores of TSC1-NTD, causing a loss of 23 amino acids from K70 to G94, which disrupts the hydrophobic core and thus destabilizes TSC1 (Figure 4a,b). It has been reported in the literature that the variant c.2197C>G, situated in the tuberin-type domain of TSC2, may cause severe epilepsy in children with TSC [33]. The tuberin domain of the TSC2 protein comprises mainly alpha helices connected by loops in an arrangement similar to an alpha–alpha superhelix domain [34]. The variant c.2197C>G results in the deletion of 41 amino acids from Q699 to L741 within the tuberin-type domain of tuberin (Figure 4c,d). This probably disturbs the interaction between the two helices and adversely impacts the stability of the structure. Notably, the variant c.4966G>T is located in the important domain of TSC2, the GTPase-activating protein (GAP) domain, which induces Ras homolog enriched in brain (RHEB) deactivation and fails to stimulate the kinase activity of mTOR1, thereby impeding cell proliferation (Figure 5) [35]. TSC2 c.4966G>T causes an early stop in the TSC2 ORF (p. Asp1656Tyrfs⁣^∗^42), leading to premature truncation of the protein in the GAP domain (Figure 4e,f). This could significantly impair GAP activity. The variant c.4966G>T was identified in three patients from a single family with TSC, exhibiting typical clinical manifestations of TSC [24].

A study suggests that patients with premature truncating protein are more likely to exhibit major symptoms consistent with diagnostic criteria than those without premature protein truncations [36]. In our study, it was predicted that seven variants would introduce PTCs, resulting in truncated proteins that could lead to severe clinical phenotypes. Furthermore, according to the ACMG guidelines, we ultimately reclassified nine variants as pathogenic.

Notably, while AlphaFold provides valuable insights into the potential structures of mutated proteins, the stability of truncated proteins may be compromised, and they can be recognized and degraded by cellular quality control mechanisms such as NMD or other proteolytic pathways. Therefore, although structural predictions offer useful information, the biological significance of these truncated proteins requires further experimental validation. Our study is aimed at elucidating how mutations in the TSC gene affect splicing.

To sum up, this study verified the effect of variants on TSC gene splicing through minigene splicing assays of 13 variants. Among the 13 mutations, 10 are likely to cause aberrant splicing, and the forms of splicing disruption are complex. These mutations disrupt splicing by affecting splice site recognition, interfering with regulatory elements, or disrupting splicing factors. The study also underscores the importance of assessing the impact of variants on the TSC gene at the mRNA level. With an increasing number of patients undergoing DNA sequencing for diagnostic purposes, we need to consider that these exon mutations may also lead to aberrant splicing, thereby enhancing diagnostic accuracy and counseling for TSC patients. Furthermore, the findings from this study on TSC gene mutations and splicing impacts have significant potential for personalized diagnosis and treatment planning in TSC patients. Understanding the exact nature of the splicing defects in each patient could lead to more refined and effective therapeutic strategies, potentially improving patient outcomes and reducing adverse effects.

## Figures and Tables

**Table 1:** Characterization of variants selected from this study in the TSC1 and TSC2 genes and the results of bioinformatics analysis.

**Gene**	**Variant**	**Amino acid**	**Exon (length)**	**Location**	**BDGP**	**SpliceAI** ^**a**^	**HExoSplice** ^**b**^
TSC1	c.278T>A	p. Leu93Gln	5 (153 bp)	68	NA	0.63 (−2)	−0.9741
	c.913G>A	p. Gly305Arg	9 (176 bp)	−1	5DS 0.61→0.01	0.59 (−16)	−0.2829
	c.913G>T	p. Gly305Trp	9 (176 bp)	−1	5DS 0.61→0	0.61 (−16)	−0.0988
	c.1825G>T	p. Glu609⁣^∗^	15 (559 bp)	−173	NA	0.86 (−3)	−2.6251

TSC2	c.334C>T	p. Gln112⁣^∗^	4 (111 bp)	−3	5DS 1.0→0.97	0.96 (−2)	−1.0479
	c.774G>C	p. Lys258Asn	8 (126 bp)	−1	5DS 0.28→NA	0.90 (0)	0.2171
	c.1246G>T	p. Asp416Tyr	12 (138 bp)	−12	NA	0.65 (11)	−2.6891
	c.1255C>T	p. Pro419Ser	12 (138 bp)	−3	5DS 0.04→0.24	0.90 (2)	−0.118
	c.2197C>G	p. Leu733Val	20 (123 bp)	−24	NA	0.95 (−1)	−0.7307
	c.3131G>C	p. Arg1044Thr	27 (165 bp)	−1	5DS 0.04→NA	0.84 (0)	0.4744
	c.4493G>A	p. Ser1498Asn	34 (488 bp)	−1	5DS 0.86→0.08	0.83 (0)	0.0642
	c.4493G>C	p. Ser1498Thr	34 (488 bp)	−1	5DS 0.86→0.12	0.92 (0)	0.2683
	c.4966G>T	p. Asp1656Tyr	38 (140 bp)	−24	NA	0.82 (−6)	−2.3220

*Note:* Reference sequence: TSC1: NM_000368.5 and TSC2: NM_000548.5. Location of variants: “+” indicates distance from the 5⁣′ end of the exon, and “−” represents distance from the 3⁣′ end of the exon.

Abbreviations: ESRseq, exonic splicing regulatory sequences; NA, not applicable.

^a^SpliceAI: SpliceAI parameters were as follows: genome version hg38, score type raw, and max distance 10000 nt; SpliceAI positions are annotated as “−” if upstream of the variant or “+” if downstream.

^b^HExoSplice (ESRseq score variant vs. WT): negative ESRseq scores indicate the decrease of ESEs or the increase of ESS motifs. ESE motifs facilitate exon recognition, while the ESS motif inhibits it.

**Table 2:** Splicing outcomes for TSC1 and TSC2 gene exonic variants with impact on splicing.

**Gene/protein**	**DNA variants**	**Splicing outcome** ^**a**^	**Frameshift**	**Protein effect**	**ACMG criteria after splicing assay**	**Reclassification**
TSC1/hamartin	c.278T>A	△E5p69	In-frame deletions (codon211-279)	23aa loss in TSC1-NTD	PVS1, PM2, PP4	VUS→P
	c.913G>A	▼E9q16	p. Gly305Argfs⁣^∗^41	Truncated protein	PVS1, PM2, PP4	VUS→P
	c.913G>T	▼E9q16	p. Gly305Trpfs⁣^∗^41	Truncated protein	PVS1, PM2, PP4	VUS→P
	c.1825G>T	No change	No change	No change	PVS1, PM2, PP3	P→P

TSC2/tuberin	c.334C>T	▼E4q90 (15.62%) △E4q4 (34.61%) △E4 (49.77%)	In-frame insertion (codon337-426) p. Gln112⁣^∗^fs⁣^∗^70 In-frame deletions (codon226-336)	30aa increase in N-terminal Truncated protein 37aa loss in N-terminal	PVS1, PM2, PP3	P→P
	c.774G>C	▼E8q27 (72.54%) △E8 (27.46%)	In-frame insertion (codon775-801) In-frame deletions (codon649-774)	9aa increase in N-terminal 42aa loss in N-terminal	?/PVS1, PM2, PP4	VUS→P
	c.1246G>T	△E12q17 (52%) △E12 (48%)	p. Asp416Tyfs⁣^∗^ In-frame deletions (codon1120-1257)	Truncated protein 46aa loss in N-terminal	PVS1, PM2, PP4/PVS1, PM2, PP4	VUS→P
	c.1255C>T	△E12q4	p. Pro419Sefs⁣^∗^6	Truncated protein	PVS1, PM2, PP4	VUS→P
	c.2197C>G	△E20	In-frame deletions (codon2098-2220)	41aa loss in tuberin-type domain	PVS1, PM2, PP4	VUS→P
	c.3131G>C	▼E27 (40.95%) △E27-28 (59.05%)	p. Arg1044Thfs⁣^∗^27 In-frame deletions (codon2967-3284)	Truncated protein 106aa loss in cytoplasmic domain	PVS1, PM2, PP4/PVS1_M, PM2, PP4	VUS→P
	c.4493G>A	No change	No change	No change	PM1, PM2, PP3	VUS→VUS
	c.4493G>C	No change	No change	No change	PM1, PM2, PP3	VUS→VUS
	c.4966G>T	△E38q29	p. Asp1656Tyrfs⁣^∗^42	Truncated protein	PVS1, PM2, PP4	VUS→P

*Note:* △, exonic deletion; ▼, intronic retention; p, the alteration of the 3⁣′ss; q, the alteration of the 5⁣′ss. The number indicates the number of nucleotides inserted or deleted.

Abbreviation: aa, amino acid.

^a^Splicing outcome: the proportion of each transcript is indicated between parentheses.

## Data Availability

The original contributions presented in the study are included in the article/supporting information. The data that support the findings of this study are available on request from the corresponding author.

## General Statement

*Web Resources*. SpliceAI: https://spliceailookup.broadinstitute.org/, BDGP: https://www.fruitfly.org/, HExoSplice: http://bioinfo.univ-rouen.fr/HExoSplice/, VulExMap: https://vulexmap.compbio.sdu.dk/, DeepCLIP: https://deepclip.compbio.sdu.dk/, PolyPhen-2: http://genetics.bwh.harvard.edu/pph2/index.shtml, MutationTaster: https://www.mutationtaster.org/, SIFT: http://provean.jcvi.org/index.php, AlphaFold 3: https://alphafoldserver.com/.

## Supporting Information

Supporting Information Additional supporting information can be found online in the Supporting Information section. Table S1: Primers for PCR amplification of exons in TSC1 and TSC2 genes selected from this study. Table S2: Mutagenesis primers of exon variants in TSC1 and TSC2 genes. Table S3: DeepCLIP analysis of TSC variants. Table S4: In silico prediction for the pathogenicity of candidate missense variants. Figure S1: Schematic illustrations of the minigene expression constructs in the context of wild-type inserts. Figure S2: Sanger sequencing results of all constructed recombinant plasmids. Figure S3: Sequencing diagrams of transcripts of variants of the TSC1 gene. Figure S4: Sequencing diagrams of transcripts for the variant c.334C>T in Exon 4 of the TSC2 gene. Figure S5: Sequencing diagrams of transcripts for the variant c.774G>C in Exon 8 of the TSC2 gene. Figure S6: Sequencing diagrams of transcripts for the variant c.4966G>T in Exon 38 of the TSC2 gene. Figure S7: Sequencing diagrams of transcripts for the variants c.1246G>T and c.1255C>T in Exon 12 of the TSC2 gene. Figure S8: Sequencing diagrams of transcripts for the variant c.2197C>G in Exon 20 of the TSC2 gene. Figure S9: Sequencing diagrams of transcripts for the variant c.3131G>C in Exons 27–28 of the TSC2 gene. Figure S10: VulExMap plot of TSC1 and TSC2 genes. Figure S11: Structural predictions for TSC gene variants. Figure S12: Complete AGAR gel image of the TSC1 gene. Figure S13: Complete AGAR gel image of the TSC2 gene.
