# Supplementary material for: Splicing Analysis of Exonic TSC1 and TSC2 Gene Variants Causing Tuberous Sclerosis Complex
Source: Hum Mutat. 2025 Apr 1;2025:1497712. doi: 10.1155/humu/1497712 (PMC11978479; doi:10.1155/humu/1497712)
Supplement: Supporting Information — Additional supporting information can be found online in the Supporting Information section. Table S1: Primers for PCR amplification of exons in TSC1 and TSC2 genes selected from this study. Table S2: Mutagenesis primers of exon variants in TSC1 and TSC2 genes. Table S3: DeepCLIP analysis of TSC variants. Table S4: In silico prediction for the pathogenicity of candidate missense variants. Figure S1: Schematic illustrations of the minigene expression constructs in the context of wild-type inserts. Figure S2: Sanger sequencing results of all constructed recombinant plasmids. Figure S3: Sequencing diagrams of transcripts of variants of the TSC1 gene. Figure S4: Sequencing diagrams of transcripts for the variant c.334C>T in Exon 4 of the TSC2 gene. Figure S5: Sequencing diagrams of transcripts for the variant c.774G>C in Exon 8 of the TSC2 gene. Figure S6: Sequencing diagrams of transcripts for the variant c.4966G>T in Exon 38 of the TSC2 gene. Figure S7: Sequencing diagrams of transcripts for the variants c.1246G>T and c.1255C>T in Exon 12 of the TSC2 gene. Figure S8: Sequencing diagrams of transcripts for the variant c.2197C>G in Exon 20 of the TSC2 gene. Figure S9: Sequencing diagrams of transcripts for the variant c.3131G>C in Exons 27–28 of the TSC2 gene. Figure S10: VulExMap plot of TSC1 and TSC2 genes. Figure S11: Structural predictions for TSC gene variants. Figure S12: Complete AGAR gel image of the TSC1 gene. Figure S13: Complete AGAR gel image of the TSC2 gene. [file 1497712.f1.docx]

Table S1 Primers for PCR amplification of exons in TSC1 and TSC2 genes selected from this study

| Gene | Exon | Forward primer (5'-3') | Reverse primer (5'-3') | Product size (bp) |
| --- | --- | --- | --- | --- |
| TSC*1* | Exon 5 | CCGCTCGAGACCCTTGCTTTACATTAGA | CTAGCTAGCTGTAACATTGAACCCATC | 519 |
| TSC*1* | Exon 9 | CCGCTCGAGATGGTCCCGCTTGTTCTT | CTAGCTAGC TTCCCTTATGAGGTAGAG | 593 |
| TSC*1* | Exon 15 | CCGCTCGAGATGCCACCCAAACTGCCTAG | CTAGCTAGC GCCTCAAGGTGGGAGTGT | 743 |
| TSC2 | Exon 4 | CCGCTCGAGCTCTGAGCCGTTCCCTGTA | CTAGCTAGCCATCCCATAAGAGGAAGGTG | 549 |
| TSC2 | Exon 8 | CCGCTCGAG TCAGGGTGGATGACAGCA | CTAGCTAGC GGCAGCAGGGTTCTCAAA | 532 |
| TSC2 | Exon 12 | CCGCTCGAG TACGGGCAGGAACAGCAG | CTAGCTAGCGGGAGGAAACTGATGGGTG | 635 |
| TSC2 | Exon 20 | CCGCTCGAG ATAGCCCTTGACGCTGTGC | CTAGCTAGC GGGAGTGCCTGCCAAGTAA | 552 |
| TSC2 | Exon 27 | CCGCTCGAG GTCTTTCCGAGCGAGGTCC | CTAGCTAGC CGCACAGGGTGGACTTAG | 662 |
| TSC2 | Exon 34 | CCGCTCGAGTGGGATGGAGGACAGATAGGG | CTAGCTAGC TAGGGCACAGGCGAGGAC | 698 |
| TSC2 | Exon 38 | CCGCTCGAG AGGTTCCGAGCCTAACAGCG | CTAGCTAGCAGACAGGAAGCACCAACTACAC | 645 |

| Gene | Variant | Forward primer (5'-3') | Reverse primer (5'-3') |
| --- | --- | --- | --- |
| TSC*1* | c.278T>A | ATCCTCTCGTTACAGGGTCATGTCAT | ATGACATGACCCTGTAACGAGAGGAT |
| TSC*1* | c.913G>A | CACAGAATAGCTATAGTAAAAAGTGTC | GACACTTTTTACTATAGCTATTCTGTG |
| TSC*1* | c.913G>T | CACAGAATAGCTATTGTAAAAAGTGTC | GACACTTTTTACAATAGCTATTCTGTG |
| TSC1 | c.1825G>T | CATCTTTTTTAGGTGGCATTGC | GCAATGCCACCTAAAAAAGATG |
| TSC2 | c.334C>T | ATCGTGCAGGGGTAGGTAAGGCC | GGCCTTACCTACCCCTGCACGAT |
| TSC2 | c.774G>C | AGCCTTGCTGGAACGTGGGGTTTCT | AGAAACCCCACGTTCCAGCAAGGCT |
| TSC2 | c.1246G>T | AGAGATGTGCGGACCAGAGGCCTGT | ACAGGCCTCTGGTACGCACATCTCT |
| TSC2 | c.1255C>T | GGACCAGAGGTCTGTGAGACC | GGTCTCACAGACCTCTGGTCC |
| TSC2 | c.2197C>G | AGTGTGGACCAGGTGTGCTCTGCT | AGCAGAGCACACCTGGTCCACACT |
| TSC2 | c.3131G>C | TGTCCCGAAGACGTCCAGGCGGCA | TGCCGCCTGGACGTCTTCGGGACA |
| TSC2 | c.4493G>A | ATCAACCCCAAGTGGGCCTCTT | AAGAGGCCCACTTGGGGTTGAT |
| TSC2 | c.4493G>C | ATCAACCCCACGTGGGCCTCTT | AAGAGGCCCACGTGGGGTTGAT |
| TSC2 | c.4966G>T | ACTCCGGTGAGTACTTCAAGCTTG | CAAGCTTGAAGTACTCACCGGAGT |

TABLE S2 Mutagenic primers for exon variants of TSC1 and TSC2 genes

TABLE S3 DeepCLIP analysis of TSC splicing variants

| Splicing Factor | Model id | Wildtype sequence | WT-score | Variant | Variant sequence | Variant score | ∆ score^1^ |
| --- | --- | --- | --- | --- | --- | --- | --- |
|  |  |  |  |  |  |  |  |
| TSC1 | | | | | | | |
| SRSF1 | RNCMPT00107_RNCMPT | GTTACTGGGTCA | 0.29 | c.278T>A | GTTACAGGGTCA | 0.62 | 0.33 |
| SRSF1 | RNCMPT00108_RNCMPT | GTTACTGGGTCA | 0.56 | c.278T>A | GTTACAGGGTCA | 0.86 | 0.30 |
| HNRNPA2B1 | RNCMPT00024_RNCMPT | GTTACTGGGTCA | 0.23 | c.278T>A | GTTACAGGGTCA | 0.92 | 0.69 |
| HNRNPL | RNCMPT00027_RNCMPT | GTTACTGGGTCA | 0.17 | c.278T>A | GTTACAGGGTCA | 0.81 | 0.64 |
| HNRNPA1 | RNCMPT00022_RNCMPT | GTTACTGGGTCA | 0.59 | c.278T>A | GTTACAGGGTCA | 0.89 | 0.30 |
| SRSF2 | RNCMPT00072_RNCMPT | TTTTTGAGGTGG | 0.60 | c.1825G>T | TTTTTTAGGTGG | 0.21 | -0.39 |
| HNRNPA2B1 | RNCMPT00024_RNCMPT | TTTTTGAGGTGG | 0.18 | c.1825G>T | TTTTTTAGGTGG | 0.74 | 0.56 |
| TSC2 | | | | | | | |
| SRSF2 | RNCMPT00072_RNCMPT | GTGCAGGGGCAG | 0.15 | c.334C>T | GTGCAGGGGTAG | 0.93 | 0.78 |
| HNRNPA1 | RNCMPT00022_RNCMPT | CCTTGCTGGAAG | 0.48 | c.774G>C | CCTTGCTGGAAC | 0.14 | -0.34 |
| HNRNPA1 | RNCMPT00023_RNCMPT | CCTTGCTGGAAG | 0.51 | c.774G>C | CCTTGCTGGAAC | 0.19 | -0.32 |
| SRSF1 | RNCMPT00106_RNCMPT | GCGGACCAGAGGCCT | 0.99 | c.1246G>T | GCGTACCAGAGGCCT | 0.18 | -0.81 |
| SRSF1 | RNCMPT00110_RNCMPT | GCGGACCAGAGGCCT | 0.90 | c.1246G>T | GCGTACCAGAGGCCT | 0.38 | -0.52 |
| SRSF1 | SRSF1_GP | GCGGACCAGAGGCCT | 0.58 | c.1246G>T | GCGTACCAGAGGCCT | 0.28 | -0.30 |
| SRSF9 | SRSF9_ENCODE-HepG2 | GCGGACCAGAGGCCT | 0.85 | c.1246G>T | GCGTACCAGAGGCCT | 0.56 | -0.29 |
| SRSF7 | RNCMPT00073_RNCMPT | GCGGACCAGAGGCCT | 0.93 | c.1246G>T | GCGTACCAGAGGCCT | 0.48 | -0.45 |
| HNRNPA2B1 | RNCMPT00024_RNCMPT | GCGGACCAGAGGCCT | 0.76 | c.1246G>T | GCGTACCAGAGGCCT | 0.23 | -0.53 |
| HNRNPL | RNCMPT00091_RNCMPT | GCGGACCAGAGGCCT | 0.08 | c.1246G>T | GCGTACCAGAGGCCT | 0.90 | 0.82 |
| HNRNPL | RNCMPT00091_RNCMPT | CCAGCTGTGCTC | 0.33 | c.2197C>G | CCAGGTGTGCTC | 0.91 | 0.58 |
| HNRNPA1 | RNCMPT00023_RNCMPT | CCAGCTGTGCTC | 0.10 | c.2197C>G | CCAGGTGTGCTC | 0.53 | 0.43 |
| SRSF1 | SRSF1_GP | TGTCCCGAAGAG | 0.37 | c.3131G>C | TGTCCCGAAGAC | 0.69 | 0.32 |
| SRSF2 | RNCMPT00072_RNCMPT | TGTCCCGAAGAG | 0.69 | c.3131G>C | TGTCCCGAAGAC | 0.25 | -0.44 |
| HNRNPA1 | RNCMPT00022_RNCMPT | TGTCCCGAAGAG | 0.57 | c.3131G>C | TGTCCCGAAGAC | 0.17 | -0.40 |
| SRSF1 | RNCMPT00106_RNCMPT | GGTGAGGACTTC | 1.00 | c.4966G>T | GGTGAGTACTTC | 0.35 | -0.64 |
| SRSF1 | RNCMPT00107_RNCMPT | GGTGAGGACTTC | 1.00 | c.4966G>T | GGTGAGTACTTC | 0.55 | -0.45 |
| SRSF1 | RNCMPT00108_RNCMPT | GGTGAGGACTTC | 1.00 | c.4966G>T | GGTGAGTACTTC | 0.28 | -0.72 |
| SRSF1 | RNCMPT00110_RNCMPT | GGTGAGGACTTC | 0.74 | c.4966G>T | GGTGAGTACTTC | 0.30 | -0.44 |
| SRSF1 | SRSF1_GP | GGTGAGGACTTC | 0.92 | c.4966G>T | GGTGAGTACTTC | 0.48 | -0.44 |
| SRSF9 | SRSF9_ENCODE-HepG2 | GGTGAGGACTTC | 0.86 | c.4966G>T | GGTGAGTACTTC | 0.40 | -0.46 |
| HNRNPL | RNCMPT00091_RNCMPT | GGTGAGGACTTC | 0.32 | c.4966G>T | GGTGAGTACTTC | 0.92 | 0.60 |

1 Gains and losses in the binding capacity of splicing factors are shown in green and red, respectively.

The cutoff for DeepCLIP analysis is set to a reduction of 0.3 or greater in the binding score at the binding site [1].

TABLE S4 In silico prediction for the pathogenicity of candidate missense variants

| Gene | Variants | Amino acid | PolyPhen-2 | Mutation Taster | SIFT |
| --- | --- | --- | --- | --- | --- |
|  |  |  |  |  |  |
| TSC1 | c.278T>A | p. Leu93Gln | Probably damaging (0.998) | Disease causing (0.999) | Damaging (0.002) |
| TSC1 | c.913G>A | p. Gly305Arg | Benign (0.227) | Disease causing (0.999) | Tolerable (0.066) |
| TSC1 | c.913G>T | p. Gly305Trp | Probably damaging (0.954) | Disease causing (0.999) | Damaging (0.001) |
| TSC2 | c.774G>C | p. Lys258Asn | Probably damaging (0.998) | Disease causing (0.999) | Tolerable (0.159) |
| TSC2 | c.1246G>T | p. Asp416Tyr | Probably damaging (0.999) | Disease causing (0.999) | Damaging (0.036) |
| TSC2 | c.1255C>T | p. Pro419Ser | Probably damaging (0.999) | Disease causing (0.999) | Damaging (0.006) |
| TSC2 | c.2197C>G | p. Leu733Val | Probably damaging (0.983) | Disease causing (0.999) | Tolerable (0.109) |
| TSC2 | c.3131G>C | p. Arg1044Thr | Probably damaging (0.942) | Disease causing (0.999) | Damaging (0.002) |
| TSC2 | c.4493G>A | p. Ser1498Asn | Probably damaging (0.817) | Disease causing (0.999) | Damaging (0.002) |
| TSC2 | c.4493G>C | p. Ser1498Thr | Probably damaging (0.572) | Disease causing (0.999) | Damaging (0.004) |
| TSC2 | c.4966G>T | p. Asp1656Tyr | Probably damaging (0.741) | Disease causing (0.999) | Damaging (0.009) |


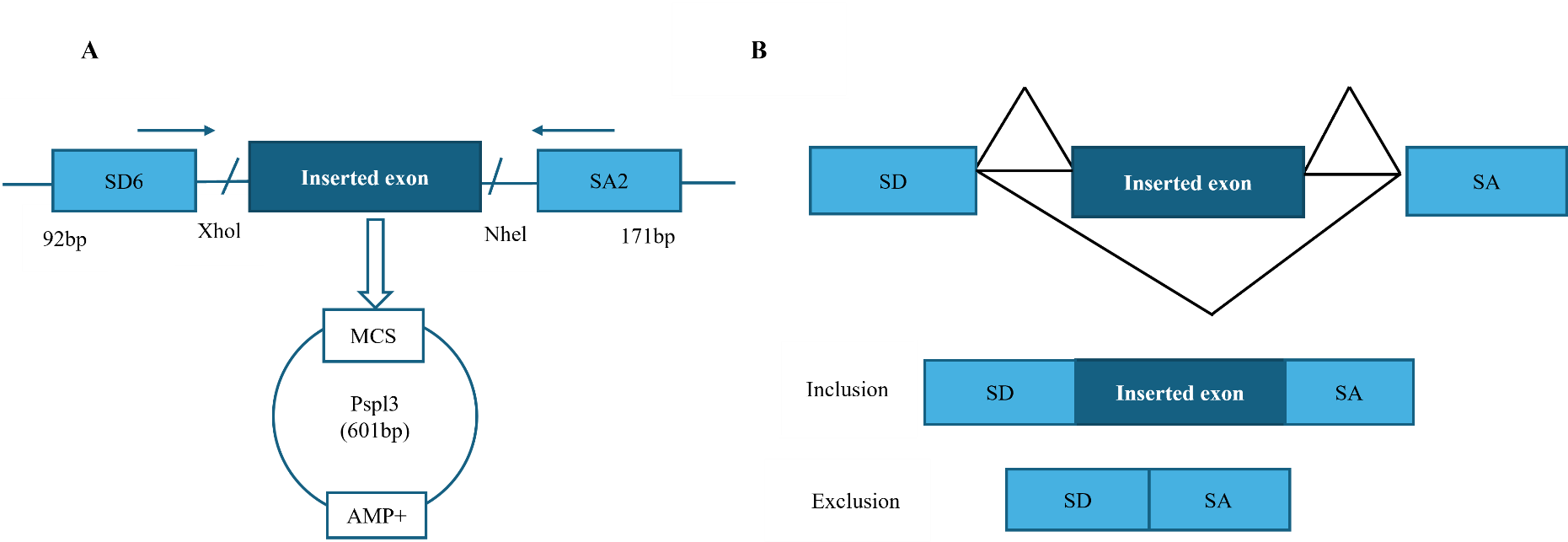


FIGURE S1 Schematic illustrations of the minigene expression constructs in the context of wild-type inserts. A The pSPL3 vector includes two exons (SD and SA) and a functional intron. The wild-type and mutant fragments of the target exon were connected to pSPL3 vector via XhoI and NheI cloning sites of pSPL3 vector, respectively, forming wild-type and mutant pSPL3 plasmids. B The transcripts with inclusion or exclusion of inserted exons of TSC gene produced by the hybrid minigene are schematically shown, lines show the splice sites used in each case. MCS, multiple cloning sites.


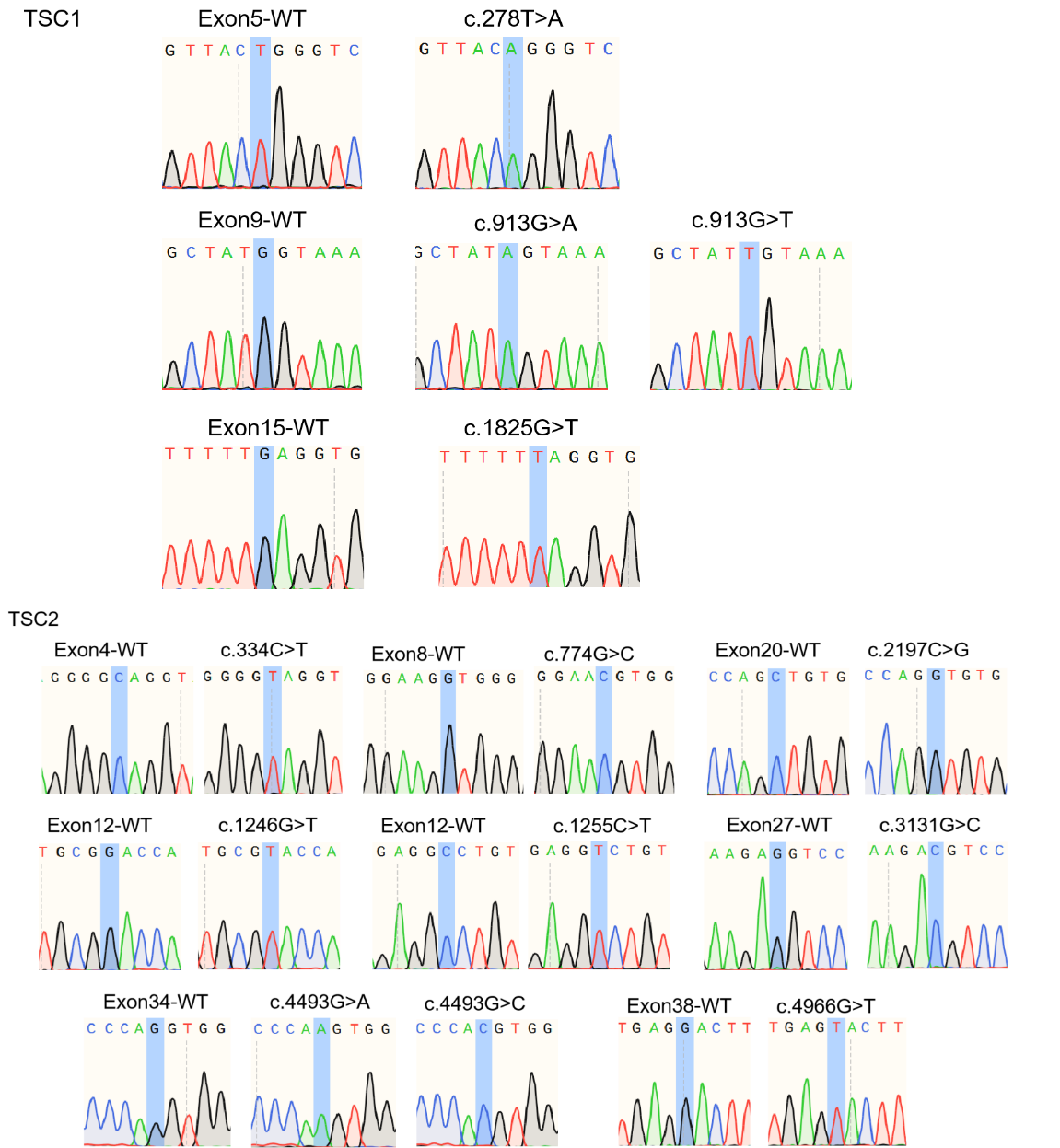


FIGURE S2 The sanger sequencing results of all constructed recombinant plasmids. The blue boxes indicate the mutation sites. The sequencing results were continuous, but the presentation was disconnected due to space limitations.

continuous, but the presentation was disconnected due to length limitation. * Indicates the variant si


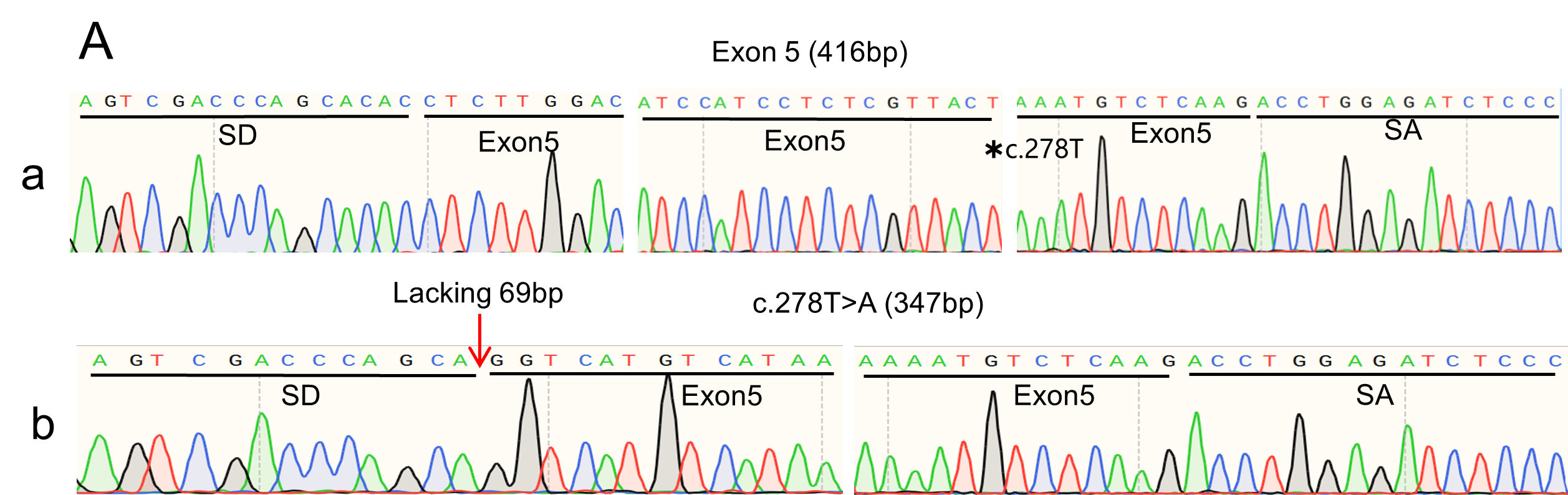


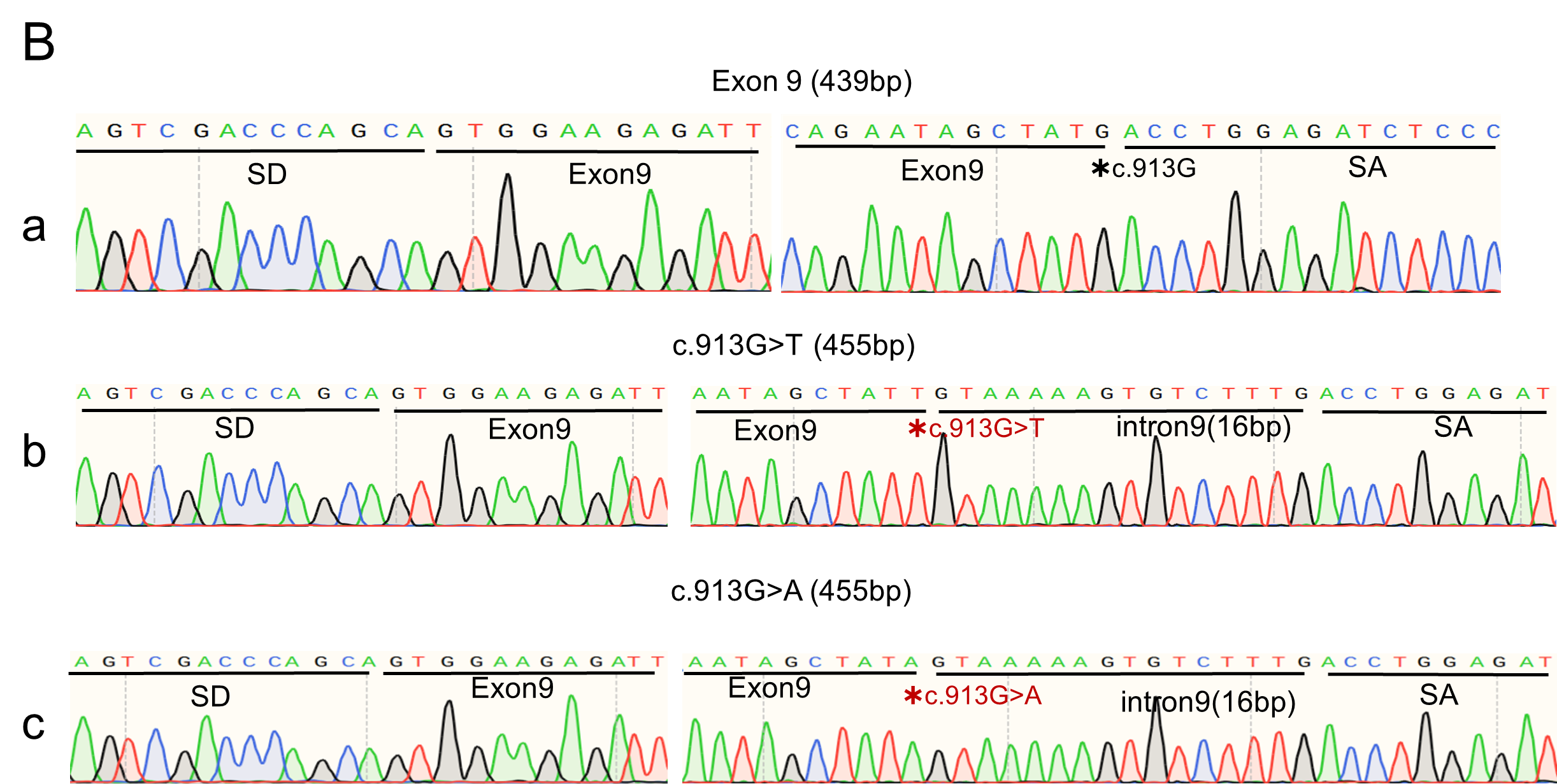


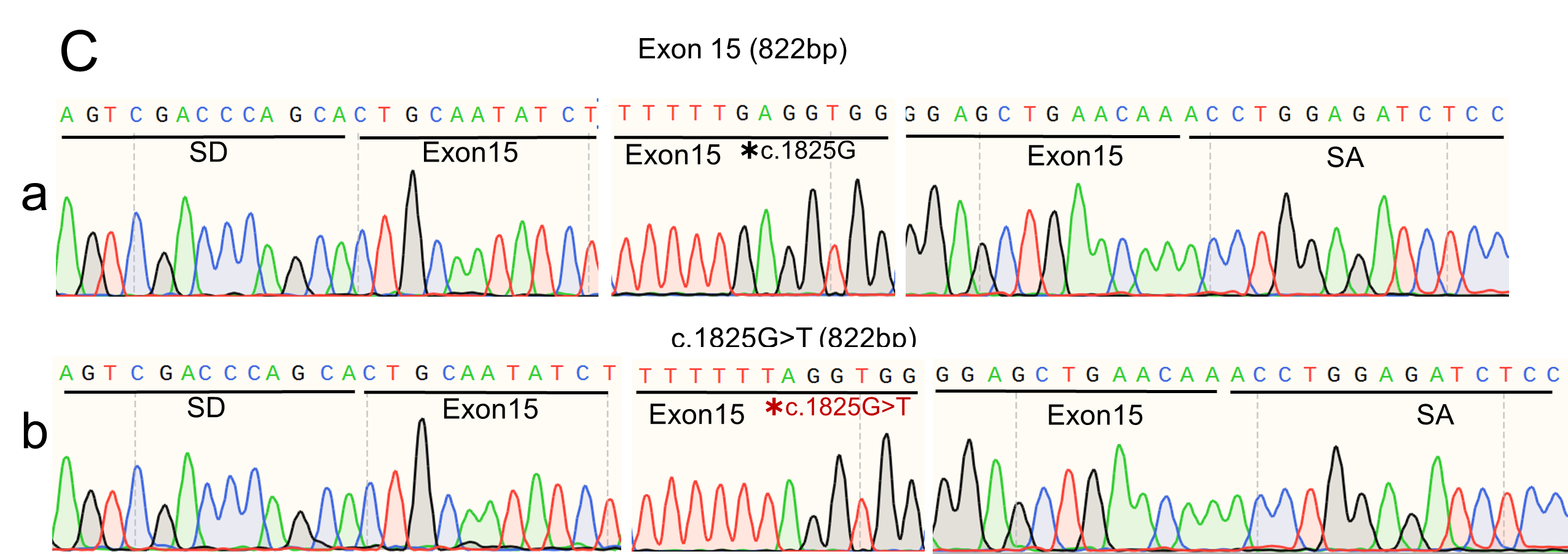


FIGURE S3 Sequencing diagrams of transcripts of variants of TSC1 gene. A Transcripts of minigene of Exon 5 and c.278T>A: (a) The fragment contained the exon5 flanked by two exons of the pSPL3 vector (SD and SA, 263bp). (b) The segment consisted of an incomplete exon 5 lacking 69-bp from the 5' end. The blue boxes indicate the mutation sites. B Transcripts of minigene of Exon 9 and c.913G>T, c.913G>A: (a) The fragment contained the exon9, SD and SA; (b) (c) The fragment contained exon 9 and partial retention (16bp) of intron 9, respectively. C Transcripts of minigene of Exon 15 and c.1825G>T: (a) (b)The fragment contained the exon 15, SD and SA. * Indicates the variant site.


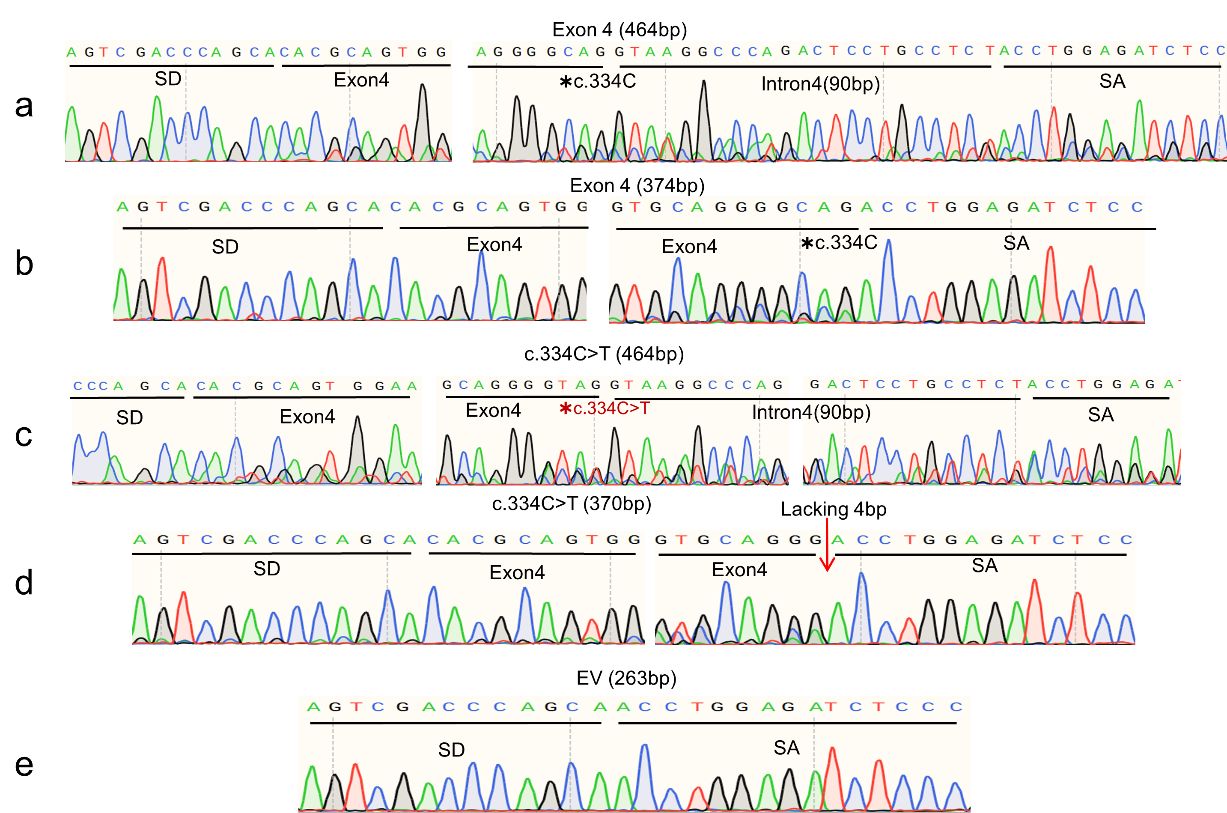


FIGURE S4 Sequencing diagrams of transcripts for the variant c.334C>T in exon 4 of TSC2 gene. (a)(c) The transcript included exon 4 and 90-bp of intron 4, SD, SA. (b) The fragment contained the exon 15, SD and SA. (d) The fragment lacked the last four bases at the 3' end of the exon 4; (e) The segment included only the exons of pSPL3. The sequencing results were continuous, but the presentation was disconnected due to space limitations; * indicates the variant site.


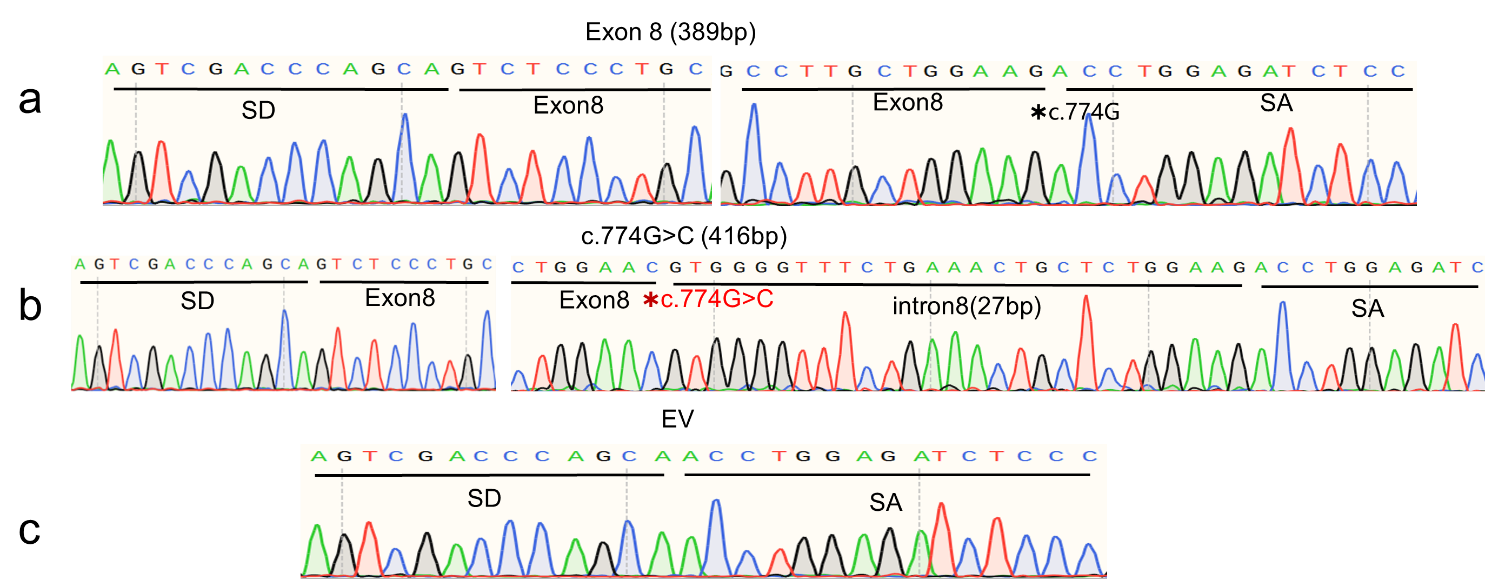


FIGURE S5 Sequencing diagrams of transcripts for the variant c.774G>C in exon 8 of TSC2 gene. (a) The fragment contained the exon 8, SD and SA; (b) The transcript included exon 8 and 27-bp of intron 8, SD, SA; (c) The segment included only SD and SA; The sequencing results were continuous, but the presentation was disconnected due to space limitations. *Indicates the variant site.


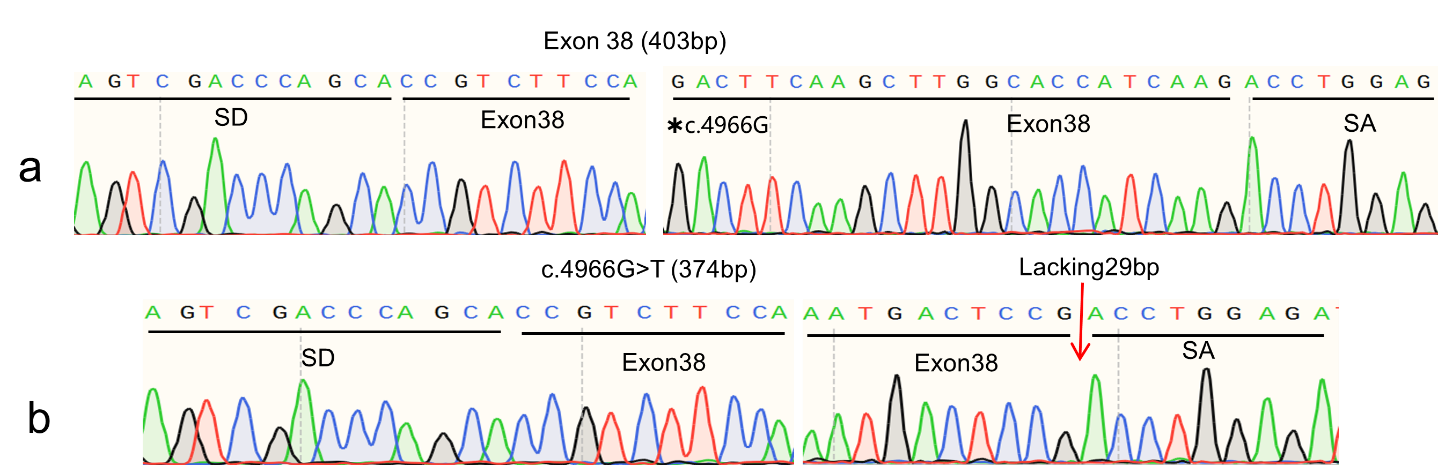


FIGURE S6 Sequencing diagrams of transcripts for the variant c.4966G>T in exon 38 of TSC2 gene. (a) The fragment contained the exon 38, SD and SA; (b) The segment consisted of an incomplete exon 38 lacking 29-bp from the3' end. The sequencing results were continuous, but the presentation was disconnected due to space limitations. * Indicates the variant site.


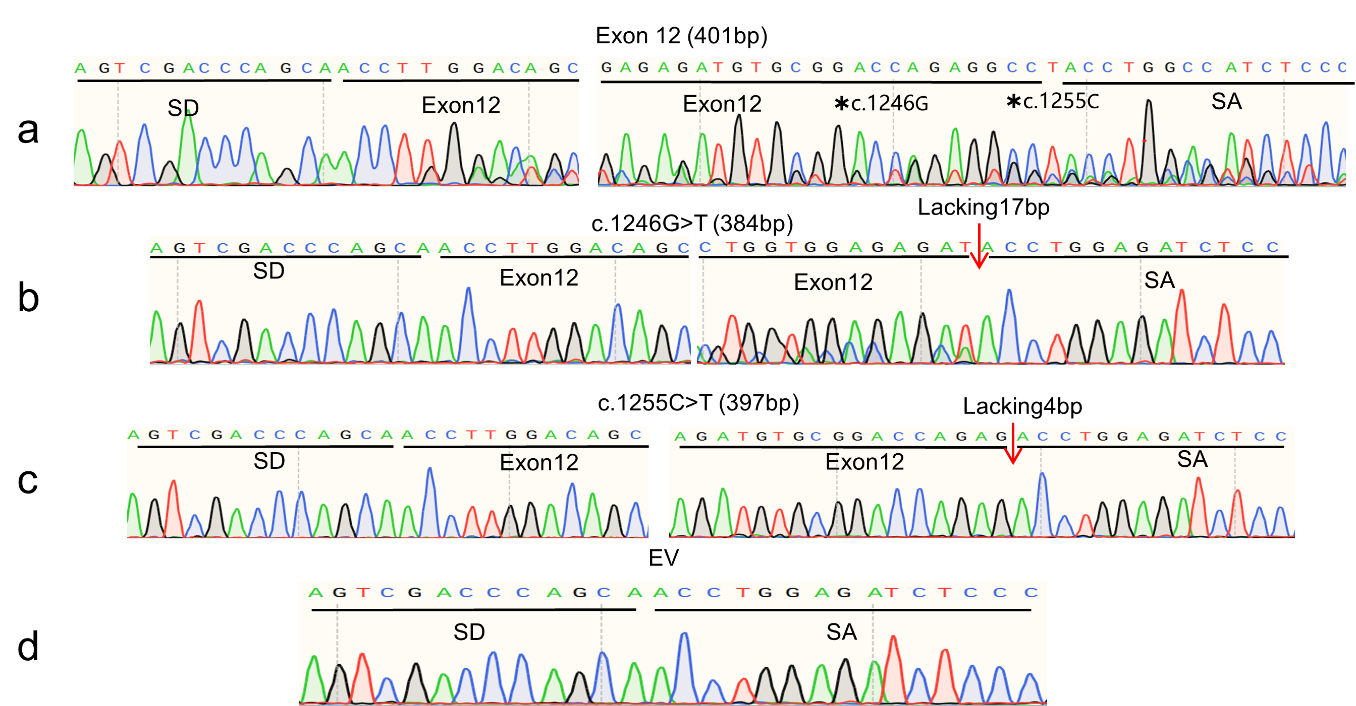


FIGURE S7 Sequencing diagrams of transcripts for the variants c.1246G>T, c.1255C>T in exon 12 of TSC2 gene. (a) The fragment contained the exon12, SD and SA; (b) The segment consisted of an incomplete exon12 lacking 17-bp from the3' end; (c) The segment consisted of an incomplete exon12 lacking 4bp from the3' end; (d) The segment consisted only SD and SA. The sequencing results were continuous, but the presentation was disconnected due to space limitations; * Indicates the variant site.


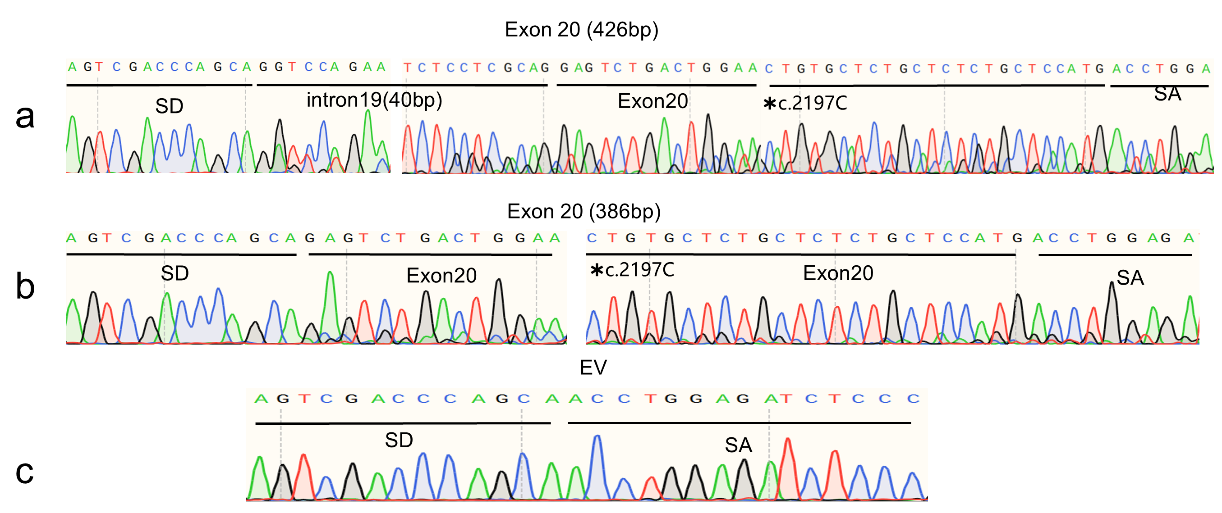


FIGURE S8 Sequencing diagrams of transcripts for the variant c.2197C>G in exon 20 of TSC2 gene. (a) Transcript encompassed the 40-bp intron 19, exon 20, SD and SA; (b) The fragment contained the exon 20, SD and SA; (c) The segment consisted only SD and SA. The sequencing results were continuous, but the presentation was disconnected due to space limitations; *Indicates the variant site.


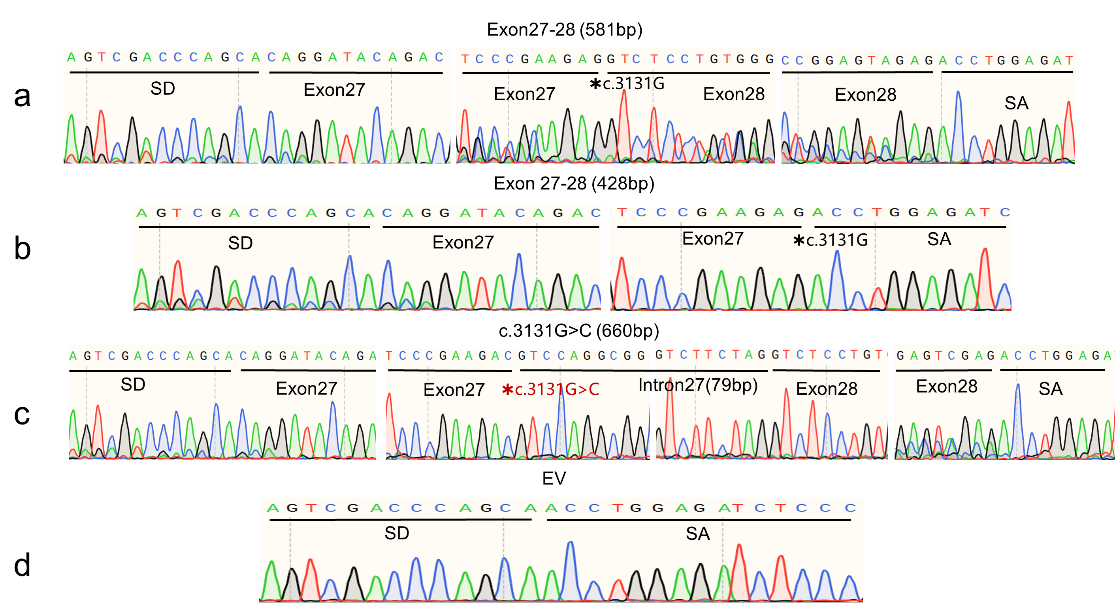


FIGURE S9 Sequencing diagrams of transcripts for the variant c.3131G>C in exon 27-28 of TSC2 gene. (a) The fragment contained the exon27-28, SD and SA; (b) The segment consisted exon 27, SD and SA; (c) Transcript include the intron 27, exon 27-28, SD and SA; (d) The segment consisted only SD and SA. The sequencing results were continuous, but the presentation was disconnected due to space limitations; * Indicates the variant site.


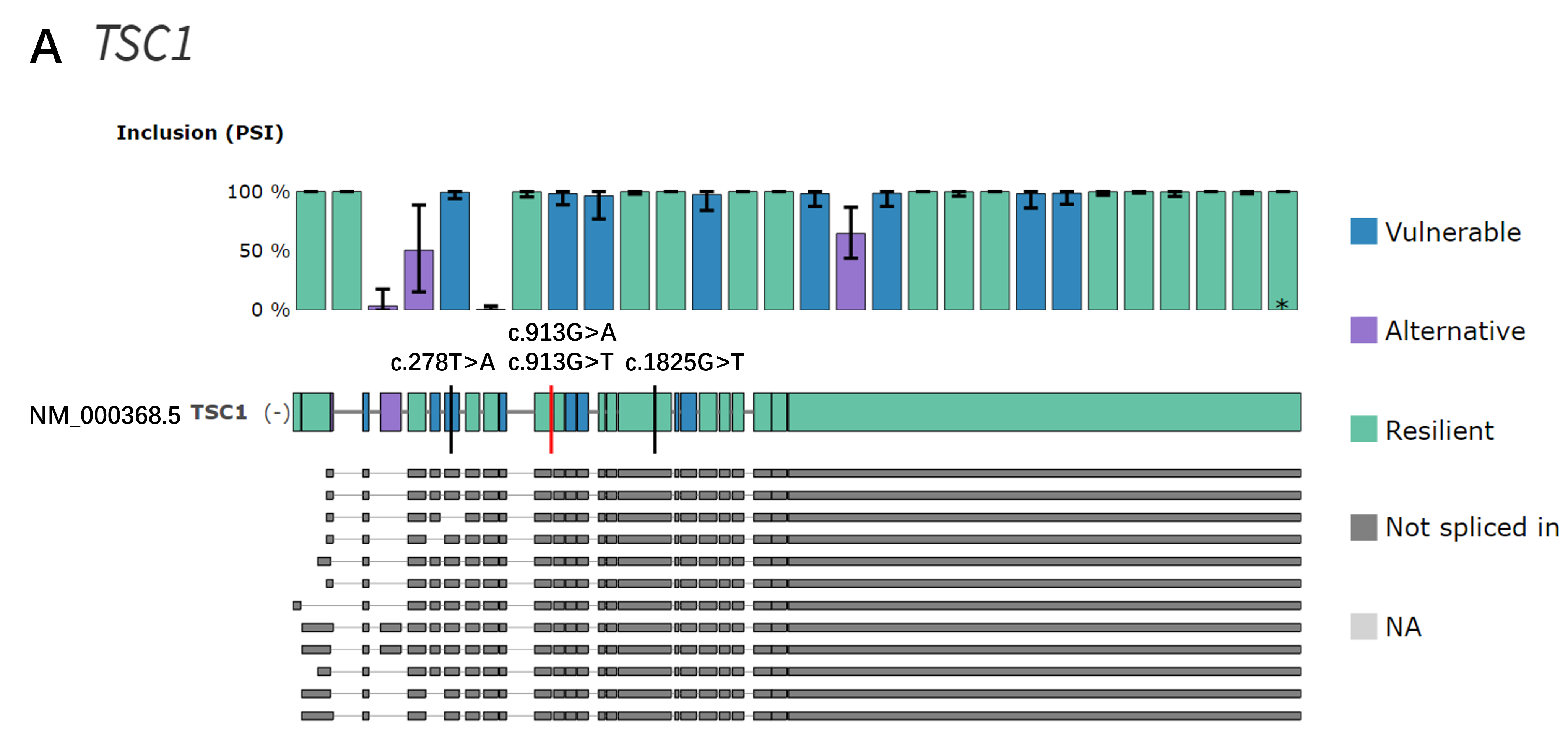


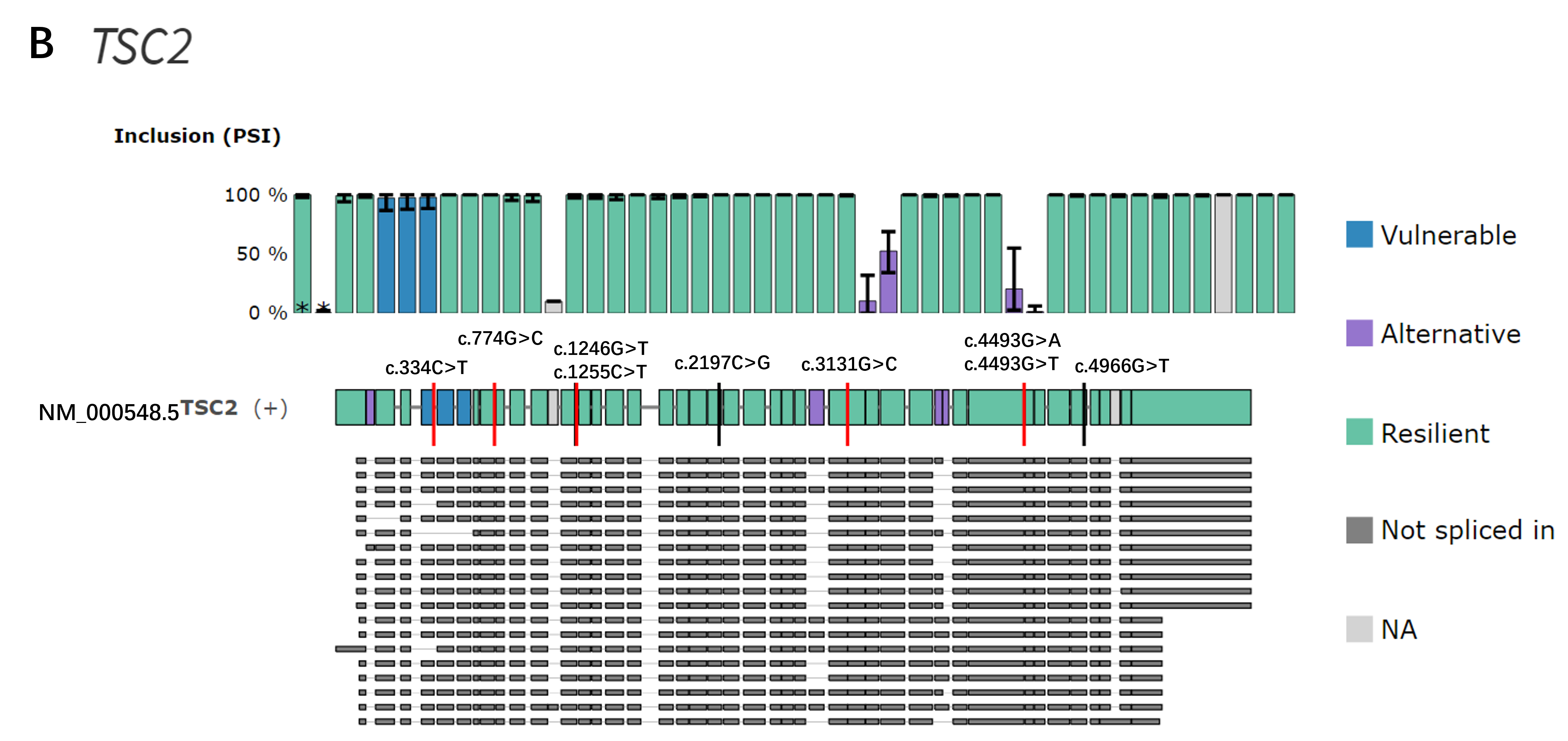


FIGURE S10 VulExMap plot of TSC1 and TSC2 genes. The bar chart shows percent spliced in (PSI) for each segment; the error bars represent the 95% inter-quantile range. Below the bar chart, a gene model is made with all segments colored by classification. Bellow the gene model are all RefSeq transcripts for the chosen gene. The top transcript, NM_000368.5 and NM_000548.5, represent the canonical protein coding transcript. A The black lines indicate that the variants c.278T>A and c.1825G>T are located in exon 5 (vulnerable) and exon 15 (resilient) of the TSC1 gene, respectively. B The black lines indicate that the variants c.1246G>T, c.2197C>G and c.4966G>T are located in exon 12(resilient), exon 20 (resilient) and exon 38 (resilient) of the TSC2 gene. If a mutation is located in the first or last 3 bp of an exon, it will be colored red. This is to indicate that the mutation is located in the splice site, and that any effect on splicing is independent of vulnerability.


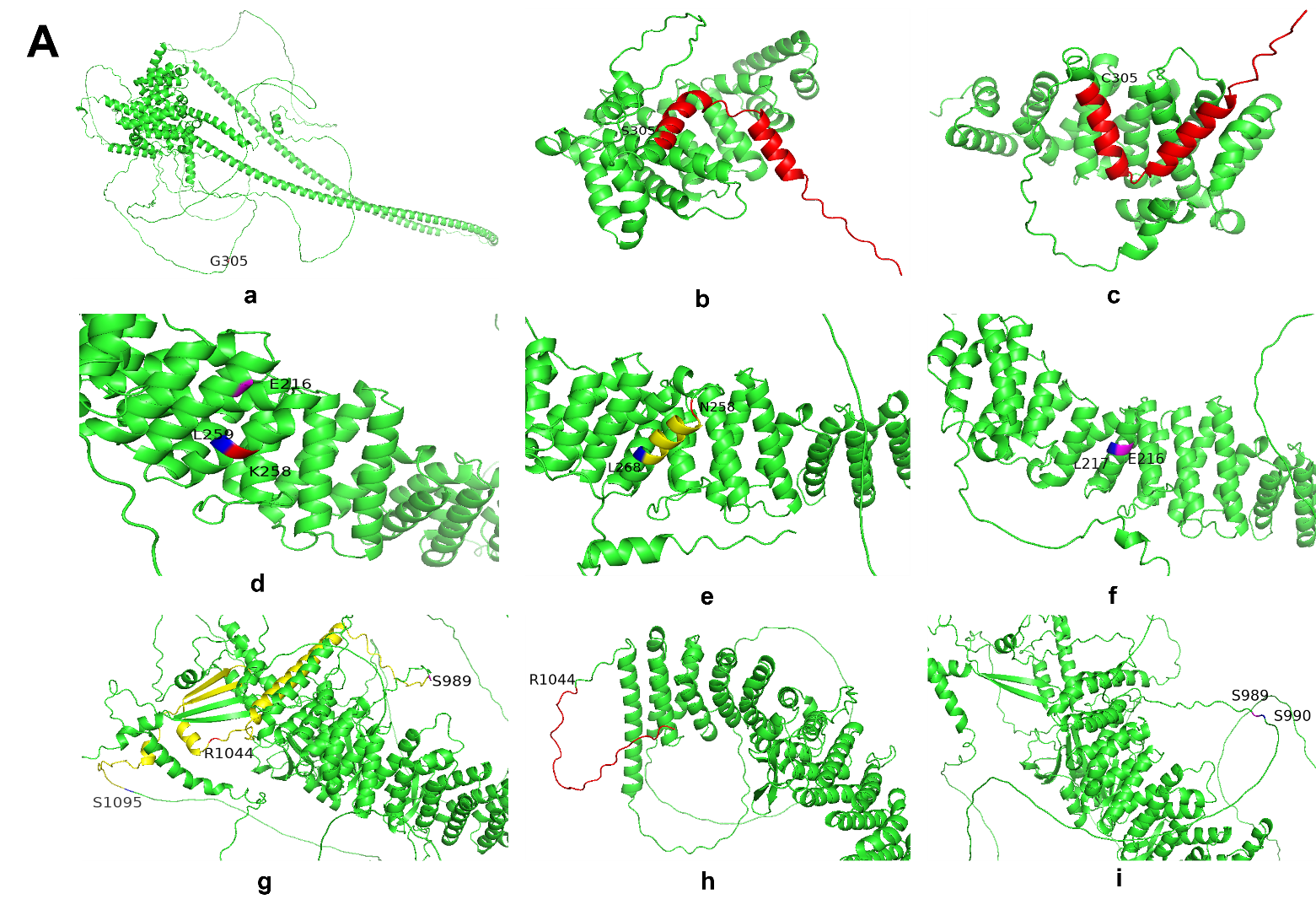


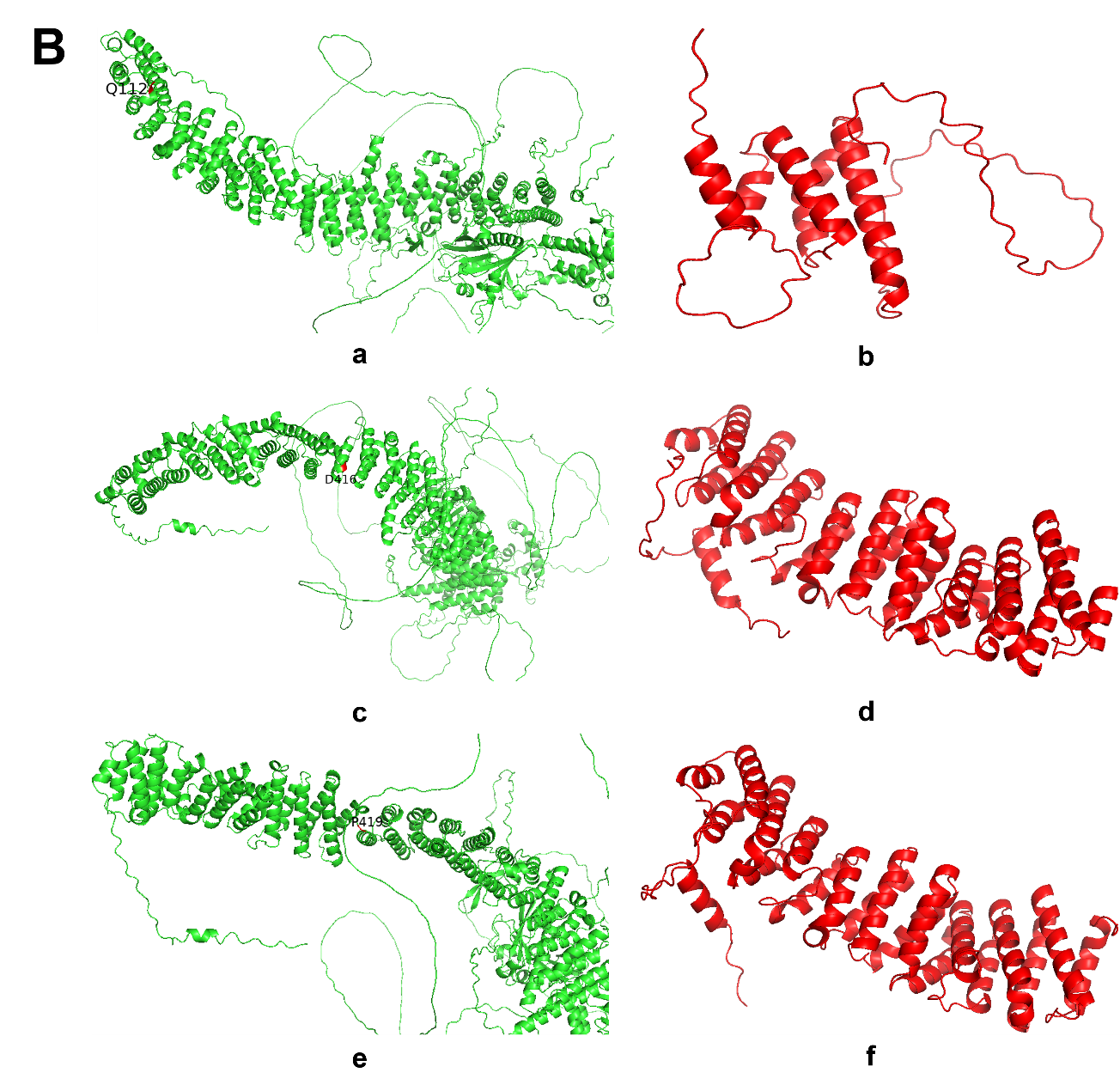


FIGURE S11 Structural predictions for TSC variants. Cartoon representation of wild type TSC (cartoon, green) in A (a, d, g) and B (a, c, e). A (a) WT TSC1, G305 is shown in red. (b)(c) The c.913G>A and c.913G>T both cause frameshift at amino acid 305, resulting in premature truncation at the 41st amino acid of the TSC2 binding domain (truncated protein is shown in red). (d) WT TSC2, E216 is shown in magenta, K258 is shown in red, and L259 is shown in blue. (e) The c.774G>C inserts 9 amino acids between N258 and L259 (N258 is shown in red, and L268 in blue, inserted amino acids are shown in yellow). (f) The c.774G>C can also cause the loss of 42 amino acids between E216 and L259 from the N-terminus of TSC2 (E216 is shown in magenta, L217 is shown in blue). (g) WT TSC2, S989 is shown in magenta, S1095 is shown in blue, R1044 is shown in red, and the amino acids between S989 and S1095 are shown in yellow. (h) The c.3131G>C results in a frameshift at amino acid 1044 and a premature truncation following the addition of 27 amino acids (the truncated protein is shown in red). (i) The c.3131G>C creates a transcript that skips exons 27 and 28, causing the loss of 106 amino acids from S989 to S1095 (S989 is shown in magenta, S990 is shown in blue). B (a), (c), (e) WT TSC2, Q112, D416, P419 are shown in red. (b), (d), (f) The variants c.334C>T, c.1246G>T, c.1255C>T result in the formation of a truncated protein in the TSC1 binding domain (truncated proteins are shown in red).


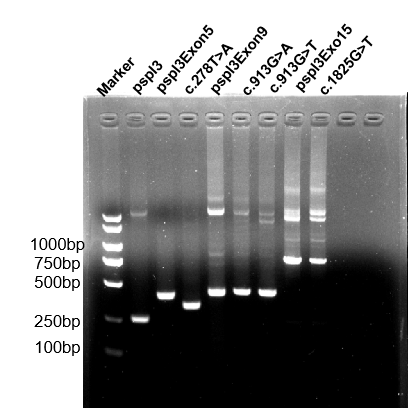


FIGURE S12 Complete AGAR gel image of TSC1 gene.


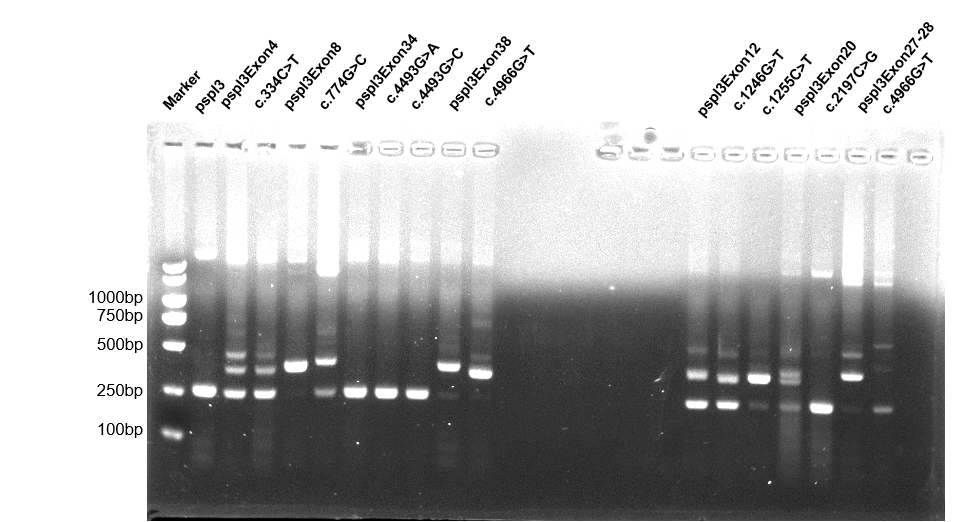


FIGURE S13 Complete AGAR gel image of TSC2 gene.

REFERENCES

[1]. Llinares-Burguet, I., et al., Splicing Dysregulation of Non-Canonical GC-5' Splice Sites of Breast Cancer Susceptibility Genes ATM and PALB2. Cancers (Basel), 2024. 16(21).
